# Green Strategies for the Preparation of Enantiomeric 5–8-Membered Carbocyclic β-Amino Acid Derivatives through CALB-Catalyzed Hydrolysis

**DOI:** 10.3390/molecules27082600

**Published:** 2022-04-18

**Authors:** Sayeh Shahmohammadi, Tünde Faragó, Márta Palkó, Enikő Forró

**Affiliations:** 1 Institute of Pharmaceutical Chemistry, Interdisciplinary Excellence Center, Faculty of Pharmacy, University of Szeged, H-6720 Szeged, Hungary; sayeh.s@pharm.u-szeged.hu (S.S.); farago.tunde@pharm.u-szeged.hu (T.F.); palko.marta@szte.hu (M.P.); 2 MTA-SZTE Stereochemistry Research Group, Hungarian Academy of Sciences, H-6720 Szeged, Hungary

**Keywords:** green strategies, enzymatic resolution, enantioselective hydrolysis, β-amino acid, ball milling

## Abstract

*Candida antarctica* lipase B-catalyzed hydrolysis of carbocyclic 5–8-membered *cis* β-amino esters was carried out in green organic media, under solvent-free and ball-milling conditions. In accordance with the high enantioselectivity factor (*E* > 200) observed in organic media, the preparative-scale resolutions of β-amino esters were performed in *t*BuOMe at 65 °C. The unreacted β-amino ester enantiomers (1*R*,2*S*) and product β-amino acid enantiomers (1*S*,2*R*) were obtained with modest to excellent enantiomeric excess (*ee*) values (*ee*_s_ > 62% and *ee*_p_ > 96%) and in good chemical yields (>25%) in one or two steps. The enantiomers were easily separated by organic solvent/H_2_O extraction.

## 1. Introduction

Interest in enantiomeric carbocyclic β-amino acids has greatly increased in recent years due to their utility in synthetic chemistry and drug research [1,2] and their pharmacological properties. For instance, both cispentacin and icofungipen exhibit antifungal activity [3,4,5,6,7,8,9]. They can be used as building blocks for the synthesis of modified peptides and self-organizing foldameric structures with increased activity and stability [8,10]. Therefore, the large number of publications about their synthesis, including those using enzymatic methods, is not surprising. As an example, an efficient direct enzymatic method through β-lactam ring cleavage was devised [11]. CALB-catalyzed hydrolysis of cyclopentane, cyclohexane, and cyclohexene skeletons bearing *cis* and *trans* β-amino esters in *i*Pr_2_O has also been published for the first time [12]. Among recent developments, implementation of green approaches enables rather attractive techniques that can carry out enantioselective reactions for the preparation of β-amino acid enantiomers. For instance, on the principle that the best solvent is no solvent, a solvent-free enzymatic method was developed through CALB-catalyzed hydrolysis of β-lactams at 70 °C to afford enantiopure β-amino acids [13]. Furthermore, in recent years, sustainable synthetic chemistry under novel mechanochemical conditions with the use of ball milling has proved to be an efficient and useful method [14,15,16,17,18,19,20]. In particular, mechanochemistry has left its mark on the road to green synthesis due to the reusability of catalysts [21,22,23,24,25,26,27]. In this regard, groundbreaking research on the concept of sustainable biocatalysis, combined with mechanochemical forces and enantioselective synthesis of biologically active molecules through mechanoenzymatic kinetic resolution of racemic compounds, has been developed [28,29,30,31,32,33]. In a noteworthy study, Perez-Venegas et al., demonstrated the employment of ball milling for liquid-assisted grinding (LAG) enzymatic resolution of *N*-benzylated-β^3^-amino esters yielding enantioenriched *N*-benzylated-β^3^-amino acids [34].

**Scheme 1:**
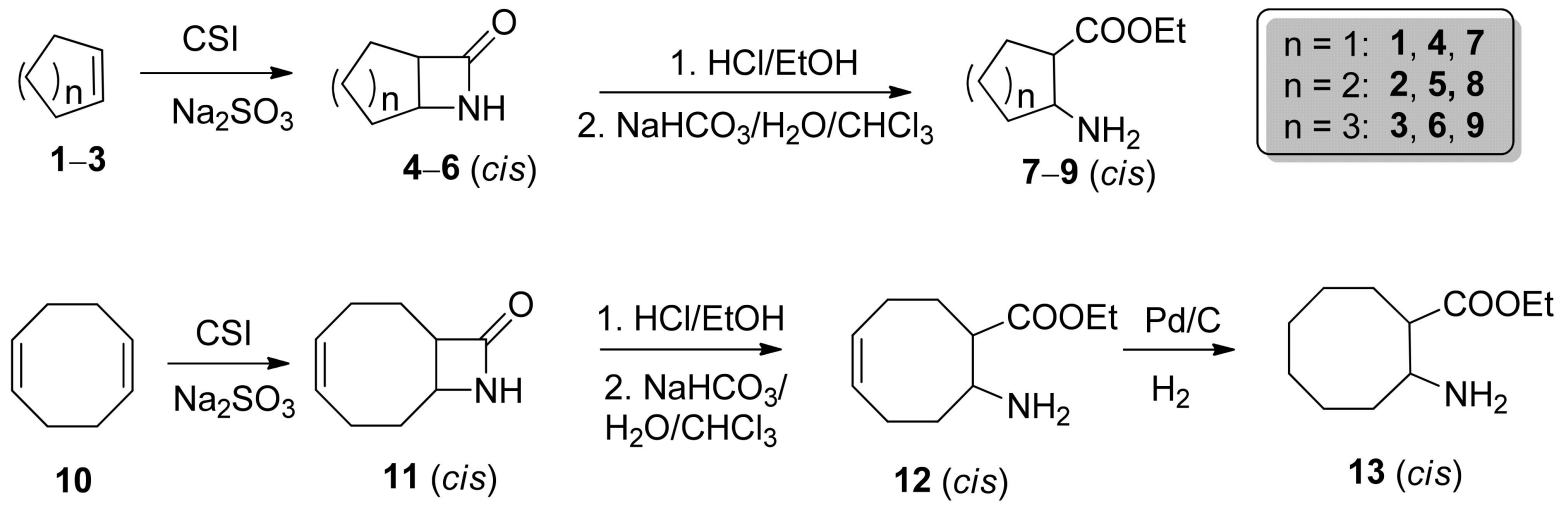
Synthesis of *cis*-amino esters **7**–**9** and **13**.

**Scheme 2:**
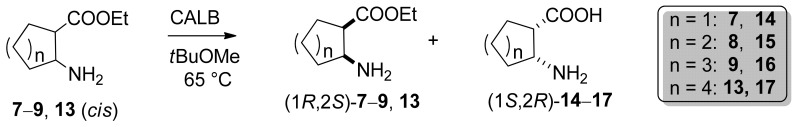
Enzymatic kinetic resolution of *cis* **7**–**9**, **13** through a hydrolytic procedure.

Herein, due to the importance of developing green methods to access enantiopure products, our primary aim was to synthesize carbocyclic 5–8-membered *cis* β-amino esters (Scheme 1). Our approach involves a comparative investigation of various environmentally friendly strategies before establishing a sustainable, CALB-catalyzed hydrolysis of *cis* amino esters **7**–**9** and **13**. The transformations deliver unreacted β-amino ester enantiomers (1*R*,2*S*)-**7**–**9**, (1*R*,2*S*)-**13**, and product β-amino acid enantiomers (1*S*,2*R*)-**14**–**17** with high enantiomeric excess (*ee*) (Scheme 2). Accordingly, reactions were planned to be carried out first in a green organic solvent, and then under solvent-free conditions. In addition, as a challenge, reactions under ball-milling conditions were planned, too.

## 2. Results and Discussion

### 2.1. Synthesis of cis-Amino Esters **7**–**9** and **13**

The 1,2-dipolar cycloaddition of chlorosylfonyl isocyanate (CSI) to cyclopentene **1**, cyclohexene **2**, cycloheptene **3** and 1,5-cyclooctadiene **10** takes place regioselectively, in accordance with the Markovnikov orientation [35], resulting in racemic *cis* β-lactams **4**, **5**, **6**, and **11**. The synthesis were performed according to known methods (slight modifications for the synthesis of **6**) [36,37,38].

Ring opening of *cis* lactams **4**–**6** with 22% ethanolic HCl furnished the desired *cis* cyclopentane, cyclohexane, cycloheptane and cyclooctane skeletons bearing amino esters **7**–**9** and unsaturated ethyl *cis*-2-aminocyclooct-5-ene carboxylate **12**. Then the latter products were reduced catalytically under H_2_ [39] to give saturated ethyl *cis*-2-aminocyclooctanecarboxylate **13** (Scheme 1).

### 2.2. Enzyme-Catalyzed Hydrolysis of Carbocyclic cis β-Amino Esters **7**–**9** and **13**

#### 2.2.1. Preliminary Experiments

**Scheme 3:**
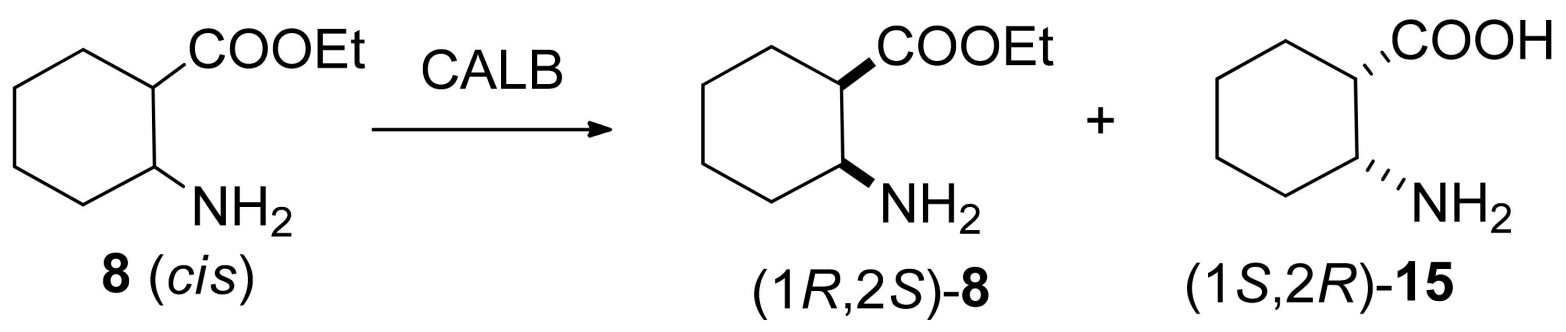
Enzymatic kinetic resolution of ethyl *cis* 2-aminocyclohexanecarboxylate **8**.

In order to determine the optimal conditions for enantioselective hydrolysis of ethyl *cis* 2-aminocyclopentanecarboxylate **7**, ethyl *cis* 2-aminocyclohexanecarboxylate **8**, ethyl *cis* 2-aminocycloheptanecarboxylate **9** and ethyl *cis* 2-aminocyclooctanecarboxylate **13** (Scheme 2), a set of preliminary experiments was performed. On the basis of earlier results achieved on the CALB-mediated enantioselective hydrolysis of 5- and 6-membered carbocyclic β-amino esters [12], the hydrolysis of the model compound ethyl *cis* 2-aminocyclohexanecarboxylate **8** (Scheme 3) was performed in *i*Pr_2_O without added H_2_O. The reaction was completed, since the H_2_O present in the reaction medium (<0.1%) or enzyme preparation (<5%) was sufficient for the hydrolysis at 65 °C (Table 1, entry 1). Several green solvents were analyzed (entries 2–6). The reaction in *t*BuOMe gave better enantioselectivity than that found in *i*Pr_2_O (conv. 39%, in both cases, *E* = 66, 133 respectively, after 8 h, entries 1, 2). The results found in propylene carbonate, 2-Me-THF, and 2-methyl-2-butanol (2M-2B) were more modest in terms of conversion and *E* (conv. 23, 12 and 6%, and *E* = 73, 74, 65 respectively, after 8 h, entries 4–6), while no reaction took place in EtOAc (entry 3). Finally, all in all, *t*BuOMe was chosen as the best green solvent for further reactions.

In order to explore the enzyme reusability, the hydrolysis of ethyl *cis* 2-aminocyclohexanecarboxylate **8** was carried out with CALB that had already been used in 1, 2 or 3 cycles (Table 2). The reaction rate was progressively decreased while the enantiomeric excess of the product appeared unaffected. This observation suggests the possibility of reusing enzyme.

In view of earlier results on β-lactam ring opening under solvent-free conditions [13], the hydrolysis of *cis* 6-membered amino ester **8** was performed in the presence of 30 mg CALB without added H_2_O. The reaction was completed without the addition of H_2_O, since the H_2_O present in enzyme preparation (<5%) was sufficient for the hydrolysis at 65 °C (Table 3, entry 2). When the reaction was carried out at room temperature (23 °C) (*E* = 45, entry 1) or at higher temperatures of 70 and 80 °C (*E* = 13, 11, entries 3, 4) a significant decrease in *E* was observed. On the basis of these data, 65 °C was selected as the optimum temperature.

When the enzyme quantity was increased from 30 mg (conv. 41% after 8 h, *E* = 70, Table 3, entry 2) to 50 mg (conv. 48% after 8 h, *E* = 177, data not shown) and 70 mg (conv. 50% after 8 h, *E* = 73, data not shown), a positive response in *E*, especially with 50 mg enzyme (substrate: enzyme ratio of 1:10), was observed. Therefore, the substrate: enzyme ratio of 1:10 was chosen for the preparative-scale, solvent-free reaction.

Inspired by results on the enzymatic hydrolysis of *N*-benzylated-β^3^-amino esters using ball milling [34], ethyl *cis* 2-aminocyclohexanecarboxylate (**8**) was hydrolyzed by using an agate jar (10 mL volume) with three agate balls (5 mm of diameter), 0.5 equiv. of added H_2_O, *t*BuOMe as a LAG (η = V (liquid; μL)/m (reagents; mg) [44], η = 2.4) at 25 Hz (Table 4, entry 1). Unfortunately, very low conversion and enantioselectivity values were observed (conv. 3% after 6 h, *E* = 6). Therefore, we started to optimize the operating frequency and found that, with decreasing frequencies, enantioselectivities increased (conv. 3, 5, 5 and 14%, and *E* = 19, 16, 21, 147, respectively after 6 h, entries 2–5). The best combination of conversion and *E* was observed at 3 Hz. When the reaction was performed with no added water at the optimized frequency with (substrate: enzyme, 1:2) and *t*BuOMe as a LAG, the catalytic activity of enzyme was not affected and enantioselectivity remained high. (*E* = 89, entry 6).

When increasing the amount of enzyme from 20 to 30 mg, both the conversion and enantioselectivity increased considerably, while reducing to 10 mg was accompanied by a drop in both conversion and *E* (Table 5, entries 1, 2 vs. Table 4, entry 5).

#### 2.2.2. Preparative-Scale Resolutions of *cis* **7**–**9**, and **13**

The preparative-scale resolution of ethyl *cis* 2-aminocyclohexanecarboxylate **8** under the optimized conditions of the investigated strategies was performed (Table 6). The resolution in *t*BuOMe was carried out in one step. However, when attempting this at larger scale, for reasons of economy, a low (substrate: enzyme 1: 4.5) ratio was employed, which maintained excellent enantioselectivity and achieved reasonable reaction time. The reaction was stopped at 50% conversion by filtering off the enzyme (entry 1). The filtered enzyme was washed with EtOAc. The solvent was evaporated to yield unreacted β-amino ester (1*R*,2*S*)-**8**. The filtered enzyme was washed with hot distilled H_2_O, then evaporation of the filtrate yielded the crystalline product β-amino acid (1*S*,2*R*)-**15**. The resolutions under solvent-free conditions (entry 2) and using ball milling (entry 3) were performed in two steps The reactions were stopped when *ee*_p_ > 96% (conv. < 50% under-run step) by adding *t*BuOMe to the reaction mixtures and filtering off the enzyme. The filtered enzyme was washed with hot distilled H_2_O. Evaporation of the filtrate yielded the crystalline product β-amino acid (1*S*,2*R*)-**15**. The repeated enzymatic reactions were stopped when *ee*_s_ > 98% (conv. > 50% over-run step). The filtered enzyme was washed with EtOAc. Evaporation of the filtrate yielded the unreacted β-amino ester (1*R*,2*S*)-**8**.

The best combination of conversion and enantioselectivity was observed in the reaction carried out in *t*BuOMe (conv. 50%, *E* > 200, after 23 h, entry 1). Therefore, preparative-scale hydrolysis of ethyl *cis* 2-aminocyclopentaneecarboxylate **7**, ethyl *cis* 2-aminocycloheptanecarboxylate **9**, and ethyl *cis* 2-aminocyclooctanecarboxylate **13** was performed in *t*BuOMe in the presence of CALB at 65 °C (Table 7). It is noteworthy that the same substrate: enzyme ratio (1: 4.5) was applicable in the large-scale hydrolysis of **7** but, due to the slow reaction rate observed in small-scale reactions, resolution of substrates with bigger cycles **9** and **13** necessitated a higher ratio of substrate: enzyme (1: 7.5). As the reactions progressed, the *ee*_p_ values of product amino acid enantiomers **14**–**17** started to decrease, while the *ee*_s_ values of unreacted esters **7**–**9** and **13** increased (data not shown). In order to obtain enantiopure amino acid products, the hydrolysis was performed in two steps, namely, once under-run (conv. < 50%) then over-run (conv. > 50%) conditions (Experimental Section).

#### 2.2.3. Determination of Absolute Configurations

The absolute configurations of ethyl (1*R*,2*S*)-2-aminocyclopentanecarboxylate **7** {[α]$\begin{array}{c} 25\\ D \end{array}$ = −6.94 (*c* 0.20 EtOH)}, ethyl (1*R*,2*S*)-2-aminocyclohexanecarboxylate **8** { [α]$\begin{array}{c} 25\\ D \end{array}$ = −11.13 (*c* 0.20 EtOH)}, ethyl (1*R*,2*S*)-2-aminocycloheptanecarboxylate **9** {[α]$\begin{array}{c} 25\\ D \end{array}$ = −4.09 (*c* 0.23 EtOH)}, ethyl (*1R*,*2S*)-2-aminocyclooctanecarboxylate **13** {[α]$\begin{array}{c} 25\\ D \end{array}$ = +20.32 (*c* 0.2 EtOH)}, (1*S*,2*R*)-2-aminocyclopentanecarboxylic acid **14** {[α]$\begin{array}{c} 25\\ D \end{array}$ = +9.41 (*c* 0.20 H_2_O), lit.[12] [α]$\begin{array}{c} 25\\ D \end{array}$ = +8 (*c* 0.23 H_2_O)}, (1*S*,2*R*)-2-aminocyclohexanecarboxylic acid **15** {[α]$\begin{array}{c} 25\\ D \end{array}$ = +19.84 (*c* 0.25 H_2_O), lit [12] [α]$\begin{array}{c} 25\\ D \end{array}$ = +21 (*c* 0.28 H_2_O)}, (1*S*,2*R*)-2-aminocycloheptanecarboxylic acid **16** [α]$\begin{array}{c} 25\\ D \end{array}$ = +6.54 (*c* 0.25 H_2_O)}, and (1*S*,2*R*)-2-aminocyclooctanecarboxylic acid **17** {[α]$\begin{array}{c} 25\\ D \end{array}$ = −19.15 (*c* 0.22 H_2_O) lit. [45] [α]$\begin{array}{c} 25\\ D \end{array}$ = −19 (*c* 0.33 H_2_O)}, were assigned by comparing the [α] values with literature data. Taking into consideration that CALB displays *S*-selective hydrolysis for the cis compounds and the analysis of GC chromatograms, the same enantio-preference for **16** was indicated.

## 3. Materials and Methods

CALB (Lipase B from *Candida antarctica*), immobilized on acrylic resin such as CSI, cycloalkenes and most of the solvents of the highest analytical grade, and sodium sulfate, anhydrous (a.r.) used as drying agent, were purchased from Sigma Aldrich (Merck KGaA Darmstadt, Germany). 2-Methyl-2-butanol (98%) was from TCI (Tokyo Chemical Industry Co., Portland, OR, USA), whereas ethyl acetate, chloroform, and acetone (a.r.) were from Novochem (Budapest, Hungary). Diethyl ether (a.r.) was from Molar Chemicals Kft (Halásztelek, Hungary). The ball-milling apparatus was Retsch 400. (Retsch GmbH, Haar, Germany). Melting points were determined with Hinotek X-4 apparatus (Hinotek, Ningbo, China) and are uncorrected. The *ee* values for the unreacted β-amino carboxylic esters and the β-amino acid enantiomers produced were determined by GC equipped with a Chirasil-l-Val column after double derivatization [40,41], with (*i*) diazomethane [Caution! the derivatization with diazomethane should be performed under a well-ventilated hood] and (*ii*) acetic anhydride in the presence of 4-dimethylaminopyridine and pyridine [80 °C for 5 min→150 °C (temperature rise 15 °C min^−1^), 15 psi]. Retention times (min) for **7**: (1*R*,2*S*) 13.887 (antipode: 14.331); for **14**: (1*S*,2*R*) 12.963 (antipode: 12.674); for **8**: (1*R*,2*S*) 16.058 (antipode: 16.316); for **15**: (1*S*,2*R*) 14.563 (antipode: 14.292); [50 °C for 5 min→140 °C (temperature rise 10 °C min^−1^), 10 psi]. For **9**: (1*R*,2*S*) 40.975 (antipode: 41.865); for **16**: (1*S*,2*R*) 35.641 (antipode: 34.869); for **13**: (1*R*,2*S*) 57.405 (antipode: 59.240); for **17**: (1*S*,2*R*) 49.309 (antipode: 48.819). Optical rotations were measured with a Jasco P 2000 Polarimeter. ^1^H- and ^13^C-NMR spectra were recorded on a Bruker Avance (Bruker Biospin, Karlsruhe, Germany) DRX 500 and 125 MHz spectrometer. The HRMS flow injection analysis was performed with Thermo Scientific Q Exactive Plus hybrid quadrupole-Orbitrap (Thermo Fisher Scientific, Waltham, MA, USA) mass spectrometer coupled to a Waters Acquity I-Class UPLCTM (Waters, Manchester, UK). (See Supplementary Materials).

### 3.1. Procedure for the Synthesis of **7**–**9** and **13**

The synthesis of racemic ethyl-2-aminocycloalkanecarboxylates **7**–**9** and **13** was carried out according to methods reported previously (the only exception is the synthesis of β-lactam **6**), starting from 50 mmol cycloalkane [35,36,37,38]. ^1^H- and ^13^C-NMR as well as HRMS data on the enantiomeric derivatives were found to be similar to those for the racemates [12,35,36,37,38,39,46,47]. Seven-membered β-lactam **6** was synthesized with a slightly modified literature procedure used for the synthesis of **4**, **5**, and **11** [38], as follows. CSI (4.42 g, 31 mmol, 1.0 equiv) was added dropwise over 60 min to neat cycloheptene (3.0 g, 31 mmol, 1.06 equiv) at 78 °C (keeping the reaction temperature as close to 78 °C as possible). After the addition was complete, the mixture was cooled to room temperature over a period of 60 min and then stirred at that temperature for 18 h. The reaction mixture was added dropwise to a stirred suspension of ice water (170 mL), Na_2_SO_3_ (17 g), and NaHCO_3_ (51 g) over a period of 20 min. The mixture was warmed to 23 °C and stirred at this temperature for 20 min followed by adding CH_2_Cl_2_ (50 mL) and stirring for an additional 5 min. The solids were collected by vacuum filtration, rinsed sequentially with water (2 × 10 mL) and CH_2_Cl_2_ (2 × 100 mL), and then discarded. The organic layer was separated from the filtrate and the aqueous layer was extracted with CH_2_Cl_2_ (3 × 25 mL). The combined organic phases were dried over Na_2_SO_4_, filtered, and were concentrated under reduced pressure to afford **6** (3.33 g, 84% yield) as a pale solid.

### 3.2. Derivatization Process

Double derivatization of β-amino acids was performed by adding a saturated solution of CH_2_N_2_ in Et_2_O dropwise to the MeOH (20 μL) aliquot until a yellow color persisted [Caution! the derivatization with diazomethane should be performed under a well-ventilated hood]. The next acylation step was carried out with Ac_2_O (15 μL) and a mixture of DMAP and pyridine (15 μL) in the same test tube, where the color immediately disappeared. Then the double-derivatized samples were analyzed by GC [40,41]. Derivatization of β-amino esters were performed in a single step by adding Ac_2_O and a mixture of DMAP/pyridine to the sample solution.

### 3.3. Procedure for the Preparative-Scale Hydrolysis of (±) cis-**5**–**8**-Membered Amino Esters

Racemic β-amino esters *cis*-**7**–**9** and *cis*-**13** (100 mg) were dissolved in *t*BuOMe (15 mL). Lipase CALB (30 mg mL^−1^ for *cis*-**7**, **8**, 50 mg mL^−1^ for *cis*-**9**, **13**) was added and the mixture was shaken in an incubator shaker at 65 °C (Table 7). The reaction for ethyl *cis* 2-aminocyclohexanecarboxylate **8** was stopped by filtering off the enzyme at 50% conversion. The filtered enzyme was washed with EtOAc (3 × 15 mL). The solvent was evaporated to yield unreacted β-amino ester (1*R*,2*S*)-**8** The filtered enzyme was washed with hot distilled H_2_O (3 × 15 mL). Evaporation of the filtrate yielded the crystalline product β-amino acid (1*S*,2*R*)-2-aminocyclohexanecarboxylic acid **15**, which was recrystallized from H_2_O/acetone. Reactions for cyclopentane, cycloheptane and cyclooctane skeletons bearing 2-amino esters *cis*-**7**, **9** and **13** were performed in two steps. When the *ee*_p_ value was >96%, the under-run reactions (conv. < 50%) were stopped by filtering off the enzyme. The filtered enzyme was washed with hot distilled H_2_O (3 × 15 mL). Evaporation of the filtrate yielded the crystalline product β-amino acids (1*S*,2*R*)-2-aminocylopentanecarboxylic acid **14**, (1*S*,2*R*)-2-aminocycloheptanecarboxylic acid **16** and (1*S*,2*R*)-2-aminocyclooctanecarboxylic acid **17**, which were recrystallized from H_2_O/acetone. In order to obtain the unreacted β-amino ester enantiomers with high *ee*, the repeated enzymatic reactions were over-run (conv. > 50%) and stopped when *ee*_s_ > 98%. The filtered enzyme was washed with EtOAc (3 × 15 mL). Evaporation of the filtrates yielded the unreacted β-amino esters (1*R*,2*S*)-**7**, **9** and (1*R,*2*S*)-**13**.

#### 3.3.1. (1*R*,2*S*)-Ethyl 2-Aminocyclopentanecarboxylate (**7**)

Yield: 31%, 0.20 mmol, brown oil, {[α]$\begin{array}{c} 25\\ D \end{array}$ = −6.94 (*c* 0.20 EtOH)}, the ^1^H-NMR spectroscopic data were similar to those in the lit. [35]. ^1^H-NMR (CDCl_3_, 500 MHz): δ = 4.16 (q, *J* = 7.1 Hz, 2H, CH_2_CH_3_), 3.60 (q, *J* = 5.9 Hz, 1H, H-2), 2.77 (m, 1H, H-1), 1.75–2.11 (m, 4H, 2 × CH_2_), 1.50–1.62 (m, 2H, CH_2_), 1.27 (t, *J* = 7.1 Hz, 3H, CH_2_CH_3_), ^13^C-NMR (CDCl_3_, 125 MHz): δ = 14.3, 22.4, 26.3, 35.0, 50.2, 54.9, 60.0, 174.0

HRMS (ESI): *m*/*z* [M + H]^+^ calcd for C_8_H_15_NO_2_: 158.11756; found: 158.11753.

#### 3.3.2. (1*R*,2*S*)-Ethyl 2-Aminocyclohexanecarboxylate (**8**)

Yield: 27%, 0.16 mmol, light yellow oil, {[α]$\begin{array}{c} 25\\ D \end{array}$ = −11.13 (*c* 0.20 EtOH)}, the ^1^H-NMR spectroscopic data were similar to those in the lit. [45]. ^1^H-NMR (CDCl_3_, 500 MHz): δ = 4.14 (q, *J* = 7.1 Hz, 2H, CH_2_CH_3_), 3.24–3.32 (m, 1H, H-2), 2.48–2.58 (m, 1H, H-1), 1.30–1.84 (m, 8H, 4 × CH_2_), 1.27 (t, *J* = 7.1 Hz, 3H, CH_2_CH_3_), ^13^C-NMR (CDCl_3_, 125 MHz): δ = 14.2, 20.9, 23.7, 24.2, 33.0, 47.4, 48.4, 59.9, 174.4.

HRMS (ESI): *m*/*z* [M + H]^+^ calcd for C_9_H_17_NO_2_: 172,13321; found: 172.13313.

#### 3.3.3. (1*R*,2*S*)-Ethyl 2-Aminocycloheptanecarboxylate (**9**)

Yield: 30%, 0.16 mmol, light brown oil, {[α]$\begin{array}{c} 25\\ D \end{array}$ = −4.09 (*c* 0.23 EtOH)}, the ^1^H-NMR (CDCl_3_, 500 MHz): δ = 4.15 (q, *J* = 7.1 Hz, 2H, CH_2_CH_3_), 3.35–3.42 (m, 1H, H-2), 2.58–2.67 (m, 1H, H-1), 1.43–1.89 (m, 12H, 6 × CH_2_), 1.27 (t, *J* = 7.1 Hz, 3H, CH_2_CH_3_), ^13^C-NMR (CDCl_3_, 125 MHz): δ = 14.21, 23.58, 24.58, 26.76, 28.41, 36.06, 50.56, 51.87, 60.06, 174.34.

HRMS (ESI): *m*/*z* [M + H]^+^ calcd for C_10_H_19_NO_2_: 186.14886; found: 186.14882.

#### 3.3.4. (1*R*,2*S*)-Ethyl 2-Aminocyclooctanecarboxylate (**13**)

Yield: 27%, 0.14 mmol, light brown oil, {[α]$\begin{array}{c} 25\\ D \end{array}$ = +20.32 (*c* 0.2 EtOH)}, the ^1^H-NMR spectroscopic data were similar to those in the lit. [38]. ^1^H-NMR (CDCl_3_, 500 MHz): δ = 4.15 (q, *J* = 7.1 Hz, 2H, CH_2_CH_3_), 3.29–3.36 (m, 1H, H-2), 2.70–2.79 (m, 1H, H-1), 1.30–1.87 (m, 14H, 7 × CH_2_), 1.27 (t, *J* = 7.1 Hz, 3H, CH_2_CH_3_), ^13^C-NMR (CDCl_3_, 125 MHz): δ =14.2, 23.3, 23.7, 25.8, 26.6, 28.1, 34.0, 47.0, 51.4, 60.2.

HRMS (ESI): *m*/*z* [M + H]^+^ calcd for C_11_H_21_NO_2_: 200.16451; found: 200.16458.

#### 3.3.5. (1*R*,2*S*)-2-Aminocyclopentanecarboxylic Acid (**14**)

Yield: 25%, 0.19 mmol, beige crystal, mp 218–222 °C, {[α]$\begin{array}{c} 25\\ D \end{array}$ = +9.41 (*c* 0.20 H_2_O), lit. [12] [α]$\begin{array}{c} 25\\ D \end{array}$ = +8 (*c* 0.23 H_2_O)}, the ^1^H-NMR spectroscopic data were similar to those in the lit. [6]. ^1^H-NMR (D_2_O, 500 MHz): δ = 3.77–3.87 (m, 1H, H-2), 2.90–3.01 (m, 1H, H-1), 2.10–2.26 (m, 2H, CH_2_), 1.73–1.99 (m, 4H, 2 × CH_2_), ^13^C-NMR (D_2_O, 125 MHz): δ = 21.3, 28.1, 29.6, 47.7, 53.1, 180.9.

HRMS (ESI): *m*/*z* [M + H]^+^ calcd for C_6_H_11_NO_2_: 130.08626; found: 130.08638.

#### 3.3.6. (1*R*,2*S*)-2-Aminocyclohexanecarboxylic Acid (**15**)

Yield: 33%, 0.23 mmol, light beige crystal, mp. 240–246 °C, {[α]$\begin{array}{c} 25\\ D \end{array}$ = +19.84 (*c* 0.25 H_2_O), lit. [12] [α]$\begin{array}{c} 25\\ D \end{array}$ = +21 (*c* 0.28 H_2_O)}, the ^1^H NMR spectroscopic data were similar to those in the lit. [12]. ^1^H-NMR (D_2_O, 500 MHz): δ = 3.50–3.61 (m, 1H, H-2), 2.67–2.79 (m, 1H, H-1), 1.40–2.09 (m, 8H, 4 × CH_2_), ^13^C-NMR (D_2_O, 125 MHz): δ = 22.1, 22.6, 26.5, 27.4, 43.6, 50.4, 180.9.

HRMS (ESI): *m*/*z* [M + H]^+^ calcd for C_7_H_13_NO_2_: 144.10191; found: 144.10197.

#### 3.3.7. (*1R*,*2S*)-2-Aminocycloheptanecarboxylic Acid (**16**)

Yield: 32%, 0.20 mmol, light beige crystal, mp. 212–216 °C, {[α]$\begin{array}{c} 25\\ D \end{array}$ = +6.54 (*c* 0.25 H_2_O)}, the ^1^H-NMR (500 MHz, D_2_O): δ = 3.72–3.80 (m, 1H, H-2), 3.15–3.24 (m, 1H, H-1), 1.52–2.18 (m, 10H, 5 × CH_2_), ^13^C-NMR (D_2_O, 125 MHz): δ = 23.2, 24.7, 26.2, 26.4, 30.1, 44.9, 52.8, 177.1.

HRMS (ESI): *m*/*z* [M + H]^+^ calcd for C_8_H_15_NO_2_: 158.11757; found: 158.11756.

#### 3.3.8. (1*R*,2*S*)-2-Aminocyclooctanecarboxylic Acid (**17**)

Yield: 28%, 0.16 mmol, light brown crystal, mp 210–216 °C, {[α]$\begin{array}{c} 25\\ D \end{array}$ = −19.15 (*c* 0.22 H_2_O) lit. [45] [α]$\begin{array}{c} 25\\ D \end{array}$ = −19 (*c* 0.33 H_2_O)}, the ^1^H-NMR (500 MHz, D_2_O): δ = 3.83–3.93 (m, 1H, H-2), 3.12–3.21 (m, 1H, H-1), 1.57–2.34 (m, 12H, 6 × CH_2_), ^13^C-NMR (D_2_O, 125 MHz): δ = 23.1, 24.6, 25.2, 25.9, 26.6, 28.8, 43.1, 51, 3, 178.0.

HRMS (ESI): *m*/*z* [M + H]^+^ calcd for C_9_H_17_NO_2_: 172.13321; found: 172.13324.

## 4. Conclusions

Efficient enzymatic strategies have been developed for the enzymatic resolution of 5–8-membered carbocyclic β-amino esters through hydrolysis in green organic media, under solvent-free conditions and using ball milling. In view of the best *E*, preparative-scale resolutions were performed in *t*BuOMe at 65 °C, resulting in the desired enantiomeric unreacted β-amino esters (1*R*,2*S*)-**7**–**9**, **13**, and product β-amino acids (1*S*,2*R*)-**14**–**17** with high *ee*_p_ values (>96%). Easy separation of the enantiomers could be achieved since the unreacted β-amino esters were soluble in organic solvent and the product β-amino acids in H_2_O. To the best of our knowledge, the lipase-catalyzed hydrolysis of 7- and 8-membered carbocyclic β-amino esters was described for the first time.

## Figures and Tables

**Table 1:** Green solvent screening in the hydrolysis of ethyl *cis* 2-aminocyclohexanecarboxylate (**8**) ^a^ in organic media.

Entry	Solvent (mL)	*ee*_s_ (%) ^b^	*ee*_p_ (%) ^c^	Conv. (%) ^d^	*E* ^e^
1	*i*Pr_2_O	60	95	39	66
2	*t*BuOMe	63	>99	39	133
3	EtOAc	-	-	-	-
4	Propylene carbonate	30	>99	23	73
5	2-Me-THF	14	>99	12	74
6	2M-2B	6	>99	6	65

^a^ 0.025 M substrate, 30 mg mL^−1^ CALB, (substrate: enzyme, 1:7), 1 mL of solvent, at 65 °C after 8 h. ^b^ According to GC after derivatization. ^c^ According to GC after double derivatization [40,41]. ^d^ *c* = *ee*_s_/(*ee*_s_ + *ee*_p_) [42]. ^e^ *E* = {*ln*[(1 − *c*) × (1 + *ee*_p_)]/*ln*[(1 − *c*) × (1 − *ee*_p_)]} [43].

**Table 2:** Catalytic activity of recycled enzyme in the hydrolysis of ethyl *cis* 2-aminocyclohexanecarboxylate (**8**) ^a^ in *t*BuOMe.

CALB (mg mL^−1^)	*ee*_s_ (%) ^b^	*ee*_p_ (%) ^c^	Conv. (%) ^d^	*E* ^e^
Once used	78	97	45	180
Twice used	58	96	37	82
3 times used	43	96	31	71

^a^ 0.025 M substrate, 30 mg mL^−1^ CALB, (substrate: enzyme, 1:7), 1 mL of *t*BuOMe, at 65 °C after 12 h. ^b^ According to GC after derivatization. ^c^ According to GC after double derivatization [40,41]. ^d^ *c* = *ee*_s_/(*ee*_s_ + *ee*_p_) [42]. ^e^ *E* = {ln[(1 − *c*) × (1 + *ee*_p_)]/ln[(1 − *c*) × (1 − *ee*_p_)]} [43].

**Table 3:** Temperature screening in the hydrolysis of ethyl *cis* 2-aminocyclohexanecarboxylate (**8**) ^a^ under solvent-free conditions.

Entry	Temp (°C)	*ee*_s_ (%) ^b^	*ee*_p_ (%) ^c^	Conv. (%) ^d^	*E* ^e^
1	23	11	95	11	45
2	65	66	94	41	70
3	70	72	72	50	13
4	80	91	57	62	11

^a^ 5 mg substrate, 30 mg CALB, (substrate: enzyme, 1:6) after 8 h. ^b^ According to GC after derivatization. ^c^ According to GC after double derivatization [40,41]. ^d^ *c* = *ee*_s_/(*ee*_s_ + *ee*_p_) [42]. ^e^ *E* = {*ln*[(1 − *c*) × (1 + *ee*_p_)]/*ln*[(1 − *c*) × (1 − *ee*_p_)]} [43].

**Table 4:** Frequency screening in the hydrolysis of ethyl *cis* 2-aminocyclohexanecarboxylate (**8**) ^a^ throughout milling.

Entry	Frequency (Hz)	*ee*_s_ (%) ^c^	*ee*_p_ (%) ^d^	Conv. (%) ^e^	*E* ^f^
1	25	2	69	3	6
2	15	3	90	3	19
3	10	5	87	5	16
4	8	5	91	5	21
5	3	15	98	14	147
6 ^b^	3	16	97	14	89

^a^ 10 mg substrate, 20 mg CALB, (substrate: enzyme, 1:2), 0.5 equiv H_2_O, 24 μL of LAG, after 6 h using ball mills. ^b^ without added H_2_O. ^c^ According to GC after derivatization. ^d^ According to GC after double derivatization [40,41]. ^e^ *c* = *ee*_s_/(*ee*_s_ + *ee*_p_) [42]. ^f^ *E* = {*ln*[(1 − *c*) × (1 + *ee*_p_)]/*ln*[(1 − *c*) × (1 − *ee*_p_)]} [43].

**Table 5:** Enzyme quantity screening in the hydrolysis of ethyl *cis* 2-aminocyclohexanecarboxylate (**8**) ^a^ throughout milling.

Entry	CALB (mg)	*ee*_s_ (%) ^b^	*ee*_p_ (%) ^c^	Conv. (%) ^d^	*E* ^e^
1	30	24	>99	20	>200
2	10	13	81	14	11

^a^ 10 mg substrate, 0.5 equiv H_2_O, 24 μL of LAG, at 3 Hz after 6 h using ball mills. ^b^ According to GC after derivatization. ^c^ According to GC after double derivatization [40,41]. ^d^ *c* = *ee*_s_/(*ee*_s_ + *ee*_p_) [42]. ^e^ *E* = {*ln*[(1 − *c*) × (1 + *ee*_p_)]/*ln*[(1 − *c*) × (1 − *ee*_p_)]} [43].

**Table 6:** Prep-scale resolution of ethyl cis 2-aminocyclohexanecarboxylate (**8**) ^a^ in *t*BuOMe, ^b^ under solvent-free and ^c^ ball milling conditions.

Entry	Rt (hours)	*ee*_s_ (%) ^d^	*ee*_p_ (%) ^e^	Conv. (%) ^f^	*E* ^g^
1 ^a^	23	96	>99	50	>200
2 ^b^	2 (22)	35 (>99)	96 (69)	27 (59)	58 (27)
3 ^c^	8 (67)	20 (98)	>99 (48)	14 (67)	163 (11)

^a^ 100 mg substrate, 30 mg mL^−1^ CALB, (substrate: enzyme, 1:4.5), 15 mL *t*BuOMe, at 65 °C, in organic media (one-step resolution). ^b^ 100 mg substrate, 1000 mg CALB, (substrate: enzyme, 1:10), at 65 °C, under solvent-free conditions (two-step resolution). ^c^ 100 mg substrate, 300 mg CALB, (substrate: enzyme, 1:3), 0.5 equiv H_2_O, 244 μL of *t*BuOMe, at 3 Hz, throughout milling (two-step resolution). ^d^ According to GC after derivatization. ^e^ According to GC after double derivatization [40,41]. ^f^ *c* = *ee*_s_/(*ee*_s_ + *ee*_p_) [42]. ^g^ *E* = {*ln*[(1 − *c*) × (1 + *ee*_p_)]/*ln*[(1 − *c*) × (1 − *ee*_p_)]} [43].

**Table 7:** CALB-catalyzed prep-scale hydrolysis of carbocyclic *cis* β-amino esters **7**–**9** and **13** in *t*BuOMe.

β-Amino Esters: (1*R*,2*S*)-7–9, 13							β-Amino Acids: (1*S*,2*R*)-14–17			
(±)	Time (hours)	Conv. (%)	Yield (%)	Isomer	*ee*_s_^e^ (%)	$[\alpha ]\begin{array}{c} 25\\ D \end{array}$ (EtOH)	Yield (%)	Isomer	*ee*_p_^f^ (%)	$[\alpha ]\begin{array}{c} 25\\ D \end{array}$ (H_2_O)
**7** ^a^	4 (24)	36 (75)	31	(1*R*,2*S*)-**7**	98	−6.94 ^g^	25	(1*S*,2*R*)-**14**	96	+9.41 ^g^
**8** ^b^	23	50	27	(1*R*,2*S*)-**8**	96	−11.13 ^g^	33	(1*S*,2*R*)-**15**	98	+19.84 ^h^
**9** ^c^	23 (3d)	20 (69)	30	(1*R*,2*S*)-**9**	91	−4.09 ^i^	32	(1*S*,2*R*)-**16**	98	+6.54 ^h^
**13** ^d^	23 (20d)	20 (62)	27	(1*R*,2*S*)-**13**	62	+20.92 ^j^	28	(1*S*,2*R*)-**17**	>99	−19.15 ^k^

^a^ 100 mg substrate, 30 mg mL^−1^ enzyme, (substrate: enzyme, 1:4.5), in 15 mL *t*BuOMe, at 65 °C. ^b^ 100 mg substrate, 30 mg mL^−1^ enzyme, (substrate: enzyme, 1:4.5), in 15 mL *t*BuOMe, at 65 °C. ^c^ 100 mg substrate, 50 mg mL^−1^ enzyme, (substrate: enzyme, 1:7.5), in 15 mL *t*BuOMe, at 65 °C. ^d^ 100 mg substrate, 50 mg mL^−1^ enzyme, (substrate: enzyme, 1:7.5), in 15 mL *t*BuOMe, at 65 °C. ^e^ According to GC after derivatization. ^f^ According to GC after double derivatization [40,41] ^g^ *c* = 0.20. ^h^ *c* = 0.25. ^i^ *c* = 0.23. ^j^ *c* = 0.19. ^k^ *c* = 0.22.

## Data Availability

The data presented in this study are available in article and Supplementary Materials.

## Supplementary Materials

The following supporting information can be downloaded at: https://www.mdpi.com/article/10.3390/molecules27082600/s1, ^1^H-NMR and ^13^C-NMR spectra of (1*R*,2*S*)-**7**–**9**, **13**, and (1*S,*2*R*)-**14**–**17**, GC chromatograms of (1*R*,2*S*)-**7**–**9**, **13**, and (1*S*,2*R*)-**14**–**17**, HRMS (ESI) spectra of (1*R*,2*S*)-**7**–**9**, **13**, and (1*S*,2*R*)-**14**–**17** can be found in supporting file.

## Sample Availability

Samples of unreacted β-amino esters (1*R*,2*S*)-**7**–**9**, **13**, and product β-amino acids (1*S*,2*R*)-**14**–**17** are not available from the authors.

## References

[1] Gardiner J., Anderson K.H., Downard A., Abell A.D. (2004). Synthesis of cyclic β-amino acid esters from methionine, allylglycine, and serine. J. Org. Chem..

[2] Bell A.D., Gardiner J. (2002). Synthesis of substituted cyclohexenyl-based β-amino acids by ring-closing metathesis. Org. Lett..

[3] Fülöp F. (2001). The chemistry of 2-aminocycloalkanecarboxylic acids. Chem. Rev..

[4] Fülöp F., Atta-ur R. (2000). The chemistry of 2-aminocyclopentanecarboxylic acid. Studies in Natural Product Chemistry.

[5] Park K., Kurth M.J. (2002). Cyclic amino acid derivatives. Tetrahedron.

[6] Mittendorf J., Benet-Buch-holz J., Fey P., Mohrs K.H. (2003). Efficient asymmetric synthesis of β-amino acid BAY 10-8888/PLD-118, a novel antifungal for the treatment of yeast infections. Synthesis.

[7] Kuhl A., Hahn M.G., Dumic M., Mittendorf J. (2005). Alicyclic beta-amino acids in medicinal chemistry. Amino Acids.

[8] Fülöp F., Martinek T.A., Tóth G.K. (2006). Application of alicyclic β-amino acids in peptide chemistry. Chem. Soc. Rev..

[9] Petraitiene R., Petraitis V., Kelaher A.M., Sarafandi A.A., Mickiene D., Groll A.H., Sein T., Bacher J., Walsh T.J. (2005). Efficacy, plasma pharmacokinetics, and safety of icofungipen, an inhibitor of *candida* isoleucyl-tRNA synthetase, in treatment of experimental disseminated candidiasis in persistently neutropenic rabbits. Antimicrob. Agents. Chemother..

[10] Steer D.L., Lew R.A., Perlmutter P., Smith A.I., Aguilar M.I. (2002). β-amino acids: Versatile peptidomimetics. Curr. Med. Chem..

[11] Forró E., Fülöp F. (2016). Cispentacin-enzymatic highlights of its 25-year history. Mini. Rev. Org. Chem..

[12] Forró E., Fülöp F. (2007). The first direct enzymatic hydrolysis of alicyclic β-amino esters: A route to enantiopure *cis* and *trans* β-amino acids. Chem. Eur. J..

[13] Forró E., Fülöp F. (2008). Vapour-assisted enzymatic hydrolysis of β-lactams in a solvent-free system. Tetrahedron Asymmetry.

[14] Hernández J.G., Bolm C. (2017). Altering product selectivity by mechanochemistry. J. Org. Chem..

[15] Rodríguez B., Rantanen T., Bolm C. (2006). Solvent-free asymmetric organocatalysis in a ball mill. Angew. Chem. Int. Ed..

[16] Declerck V., Nun P., Martinez J., Lamaty F. (2009). Solvent-free synthesis of peptides. Angew. Chem. Int. Ed..

[17] Bonnamour J., Métro T.-X., Martinez J., Lamaty F. (2013). Environmentally benign peptide synthesis using liquid-assisted ball-milling: Application to the synthesis of Leuenkephalin. Green Chem..

[18] Baig R.B.N., Varma R.S. (2012). Alternative energy input: Mechanochemical, microwave and ultrasound-assisted organic synthesis. Chem. Soc. Rev..

[19] Jones W., Eddleston M.D. (2014). Introductory lecture: Mechanochemistry, a versatile synthesis strategy for new materials. Faraday Discuss..

[20] Hernández J.G., Friščić T. (2015). Metal-catalyzed organic reactions using mechanochemistry. Tetrahedron Lett..

[21] Lawrenson S.B., Arav R., North M. (2017). The greening of peptide synthesis. Green Chem..

[22] Hernández J.G., Juaristi E. (2012). Recent efforts directed to the development of more sustainable asymmetric organocatalysis. Chem. Commun..

[23] Schmidt R., Stolle A., Ondruschka B. (2012). Aromatic substitution in ball mills: Formation of aryl chlorides and bromides using potassium peroxomonosulfate and NaX. Green Chem..

[24] Hernández J.G., Macdonald N.A.J., Mottillo C., Butler I.S., Friščić T. (2014). A mechanochemical strategy for oxidative addition: Remarkable yields and stereoselectivity in the halogenation of organometallic Re(I) complexes. Green Chem..

[25] Machuca E., Juaristi E., Ranu B., Stolle A. (2015). Ball Milling Towards Green Synthesis: Applications, Projects, Challenges.

[26] McKissic K.S., Caruso J.T., Blair R.G., Mack J. (2014). Comparison of shaking versus baking: Further understanding the energetics of a mechanochemical reaction. Green Chem..

[27] Schmidt R., Burmeister C.F., Baláž M., Kwade A., Stolle A. (2015). Effect of reaction parameters on the synthesis of 5-arylidene barbituric acid derivatives in ball mills. Org. Process Res. Dev..

[28] Venegas M.P., Juaristi E. (2021). Mechanoenzymology: State of the art and challenges towards highly sustainable biocatalysis. ChemSusChem.

[29] Bolm C., Hernandez J.G. (2018). From synthesis of amino acids and peptides to enzymatic catalysis: A bottom-up approachin mechanochemistry. ChemSusChem.

[30] Ortiz C.G.A., Venegas M.P., Caporali J.V., Juaristi E. (2019). Recent applications of mechanochemistry in enantioselective synthesis. Tetrahedron Lett..

[31] Venegas M.P., Juaristi E. (2018). Mechanoenzymatic resolution of racemic chiral amines, a green technique for the synthesis of pharmaceutical building blocks. Tetrahedron.

[32] Venegas M.P., Treviño A.M.R., Juaristi E. (2020). Dual mechanoenzymatic kinetic resolution of (±)-ketorolac. ChemCatChem.

[33] Venegas M.P., Cruz M.M.T., Feria O.S., Munguia A.L., Castillo E., Juaristi E. (2020). Thermal and mechanical stability of immobilized *Candida antarctica* lipase B: An approximation to mechanochemical energetics in enzyme catalysis. ChemCatChem.

[34] Venegas M.P., Rangel G.R., Neri A., Escalante J., Juaristi E. (2017). Mechanochemical enzymatic resolution of *N*-benzylated-β^3^-amino esters. Beilstein J. Org. Chem..

[35] Moriconi E.J., Meyer W.C. (1971). The reaction of dienes with chlorosulfonyl isocyanate. J. Org. Chem..

[36] Dragovich P.S., Murphy D.E., Dao K., Kim S.H., Li L.S., Ruebsam F., Chinh Z.S., Tran V., Xiang A.X., Zhou Y. (2008). Efficient synthesis of (1*R*,2*S*) and (1*S*,2*R*)-2-aminocyclopentanecarboxylic acid ethyl ester derivatives in enantiomerically pure form. Tetrahedron Asymmetry.

[37] Viña D., Santana L., Uriarte E., Quezada E., Valencia L. (2004). Synthesis of 1-[2-(hydroxymethyl) cyclohexyl] pyrimidine analogues of nucleosides: A comparative study. Synthesis.

[38] Palkó M., Benedek G., Forró E., Wéber E., Hänninen M., Sillanpää R., Fülöp F. (2010). Synthesis of mono- and dihydroxy-substituted 2-aminocyclooctanecarboxylic acid enantiomers. Tetrahedron Asymmetry.

[39] Forró E., Árva J., Fülöp F. (2001). Preparation of (1*R*,8*S*)-and (1*S*,8*R*)-9-azabicyclo[6.2.0]dec-4-en-10-one: Potential starting compounds for the synthesis of anatoxin-α. Tetrahedron Asymmetry.

[40] Forró E. (2009). New gas chromatographic method for the enantioseparation of β-amino acids by a rapid double derivatization technique. J. Chromatogr. A.

[41] Forró E., Fülöp F. (2010). New enzymatic two-step cascade reaction for the preparation of a key intermediate for the Taxol side-chain. Eur. J. Org. Chem..

[42] Straathof A.J.J., Rekels J.L.L., Heijnen J.J. (1995). Mass balancing in kinetic resolution: Calculating yield and enantiomeric excess using chiral balance. Biotechnol. Bioeng..

[43] Chen C.S., Fujimoto Y., Girdaukas G., Sih C.J. (1982). Quantitative analyses of biochemical kinetic resolutions of enantiomers. J. Am. Chem. Soc..

[44] Friščić T., Childs S.L., Rizvi S.A.A., Jones W. (2009). The role of solvent in mechanochemical and sonochemical cocrystal formation: A solubility-based approach for predicting cocrystallisation outcome. CrystEngComm.

[45] Forró E., Kiss L., Árva J., Fülöp F. (2017). Efficient enzymatic routes for the synthesis of new eight-membered cyclic β-amino acid and β-lactam. Molecules.

[46] Kanerva L.T., Csomós P., Sundholm O., Bernáth G., Fülöp F. (1996). Approach to highly enantiopure β-amino acid esters by using lipase catalysis in organic media. Tetrahedron Asymmetry.

[47] Forró E., Fülöp F. (2003). Lipase-catalyzed enantioselective ring opening of unactivated alicyclic-fused β-lactams in an organic solvent. Org. Lett..

