# Supplementary material for: Green Strategies for the Preparation of Enantiomeric 5–8-Membered Carbocyclic β-Amino Acid Derivatives through CALB-Catalyzed Hydrolysis
Source: Molecules. 2022 Apr 18;27(8):2600. doi: 10.3390/molecules27082600 (PMC9032184; doi:10.3390/molecules27082600)

Supplementary Information

Green Strategies for the Preparation of Enantiomeric 5–8-Membered Carbocyclic β-Amino Acid Derivatives through CALB-Catalyzed Hydrolysis

Sayeh Shahmohammadi [1,2], Tünde Faragó [1], Márta Palkó [1] and Enikő Forró [1,*]

[1] Institute of Pharmaceutical Chemistry, Interdisciplinary Excellence Center, Faculty of Pharmacy, University of Szeged, H-6720 Szeged, Hungary; sayeh.s@pharm.u-szeged.hu (S.S.);
farago.tunde@pharm.u-szeged.hu (T.F.); palko.marta@szte.hu (M.P.)
[2] MTA-SZTE Stereochemistry Research Group, Hungarian Academy of Sciences, H-6720 Szeged, Hungary
* Correspondence: forro.eniko@szte.hu; Tel.: +36-62-544964

Contents

1. ^{1}H- and ^{13}CNMR spectra of ester enantiomers (1*R*,2*S*)-**7-9** and (1*R*,2*S*)-**13** 2

2. ^{1}H- and ^{13}CNMR spectra of amino acid enantiomers (1*S*,2*R*)-**14-17** 6

3. GC Chromatograms of esters **7-9** and **13**:.. 10

4. GC Chromatograms of amino acids **14-17**: 12

5. HRMS (ESI) spectra of ester enantiomers (1*R*,2*S*)-**7-9** and (1*R*,2*S*)-**13**.......................... 14

6. HRMS (ESI) spectra of amino acid enantiomers (1*S*,2*R*)-**14-17** 18

1. ^{1}H- and ^{13}C-NMR spectra of ester enantiomers (1*R*,2*S*)-7-9 and (1*R*,2*S*)-13

Ethyl (1*R*,2*S*)-2-aminocyclopentanecarboxylate (**7**)

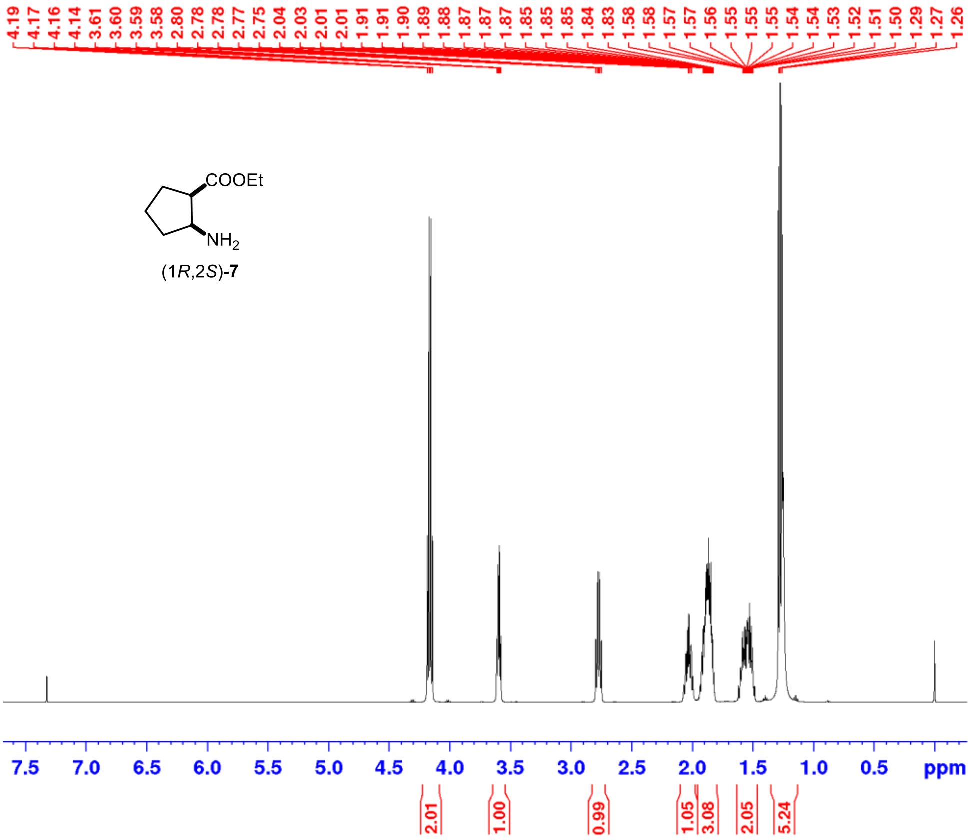

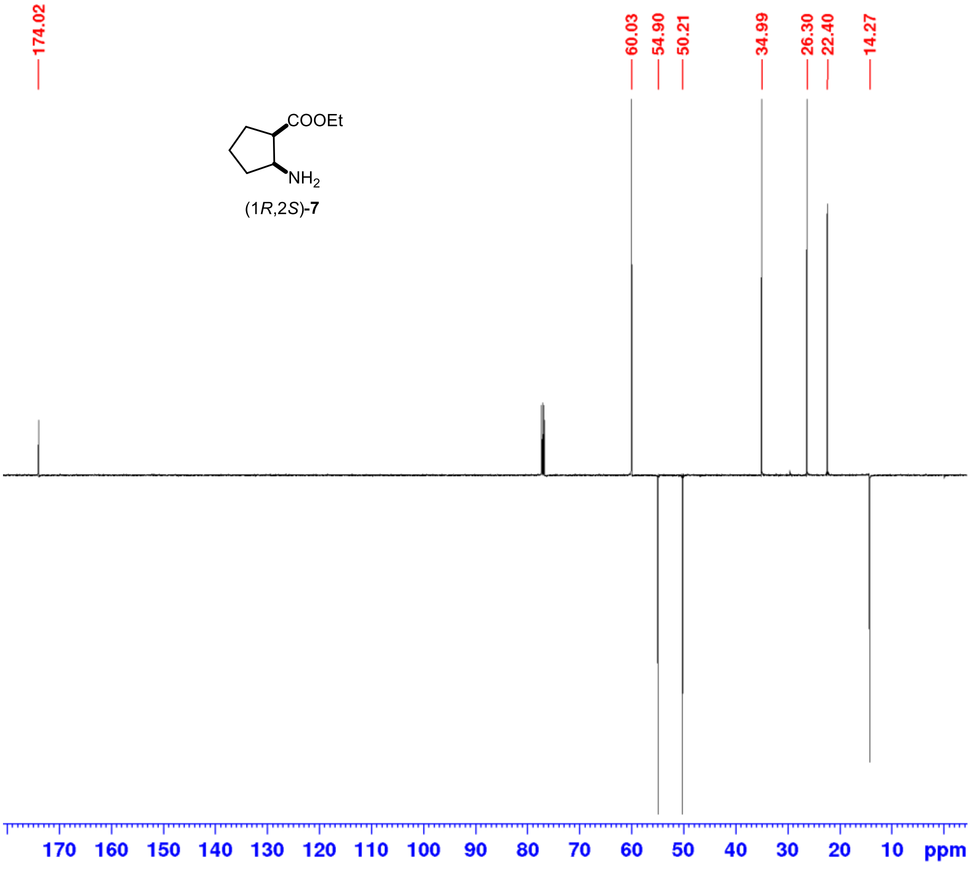

Ethyl (1*R*,2*S*)-2-aminocyclohexanecarboxylate (**8**)

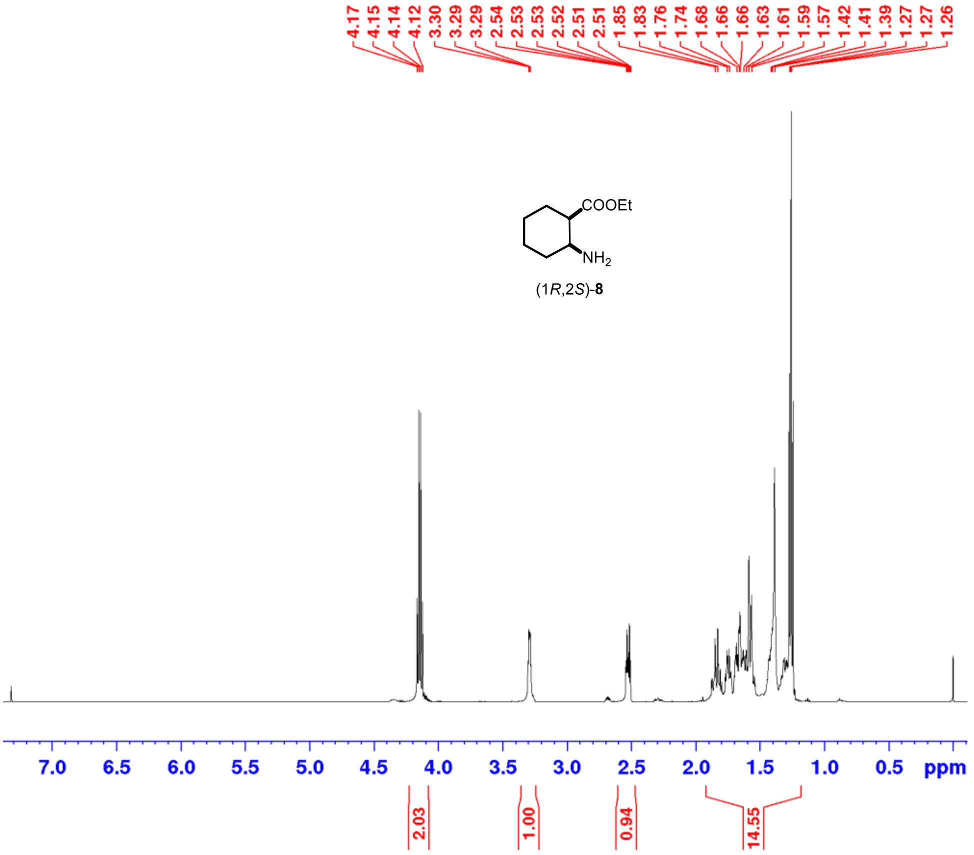

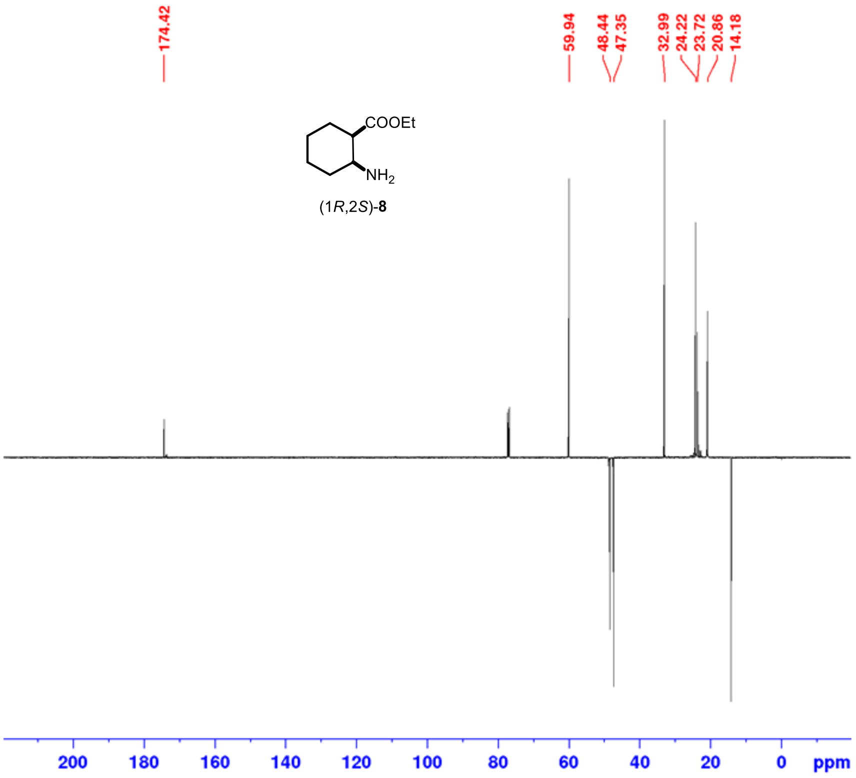

Ethyl (1*R*,2*S*)-2-aminocycloheptanecarboxylate (**9**)

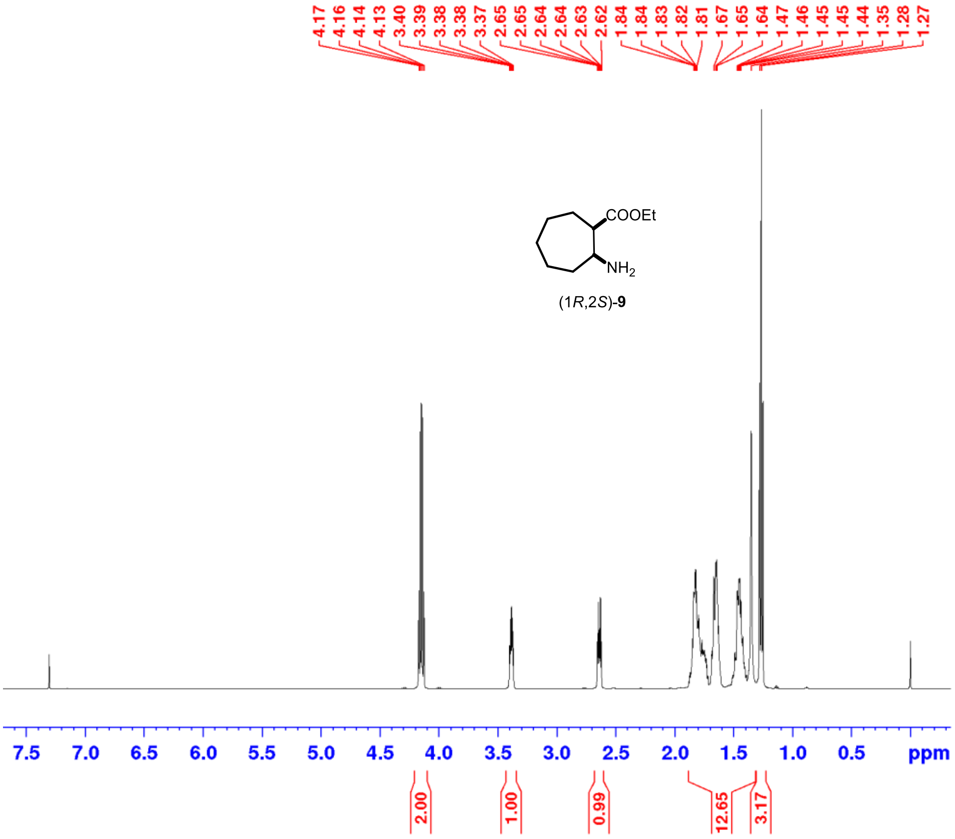

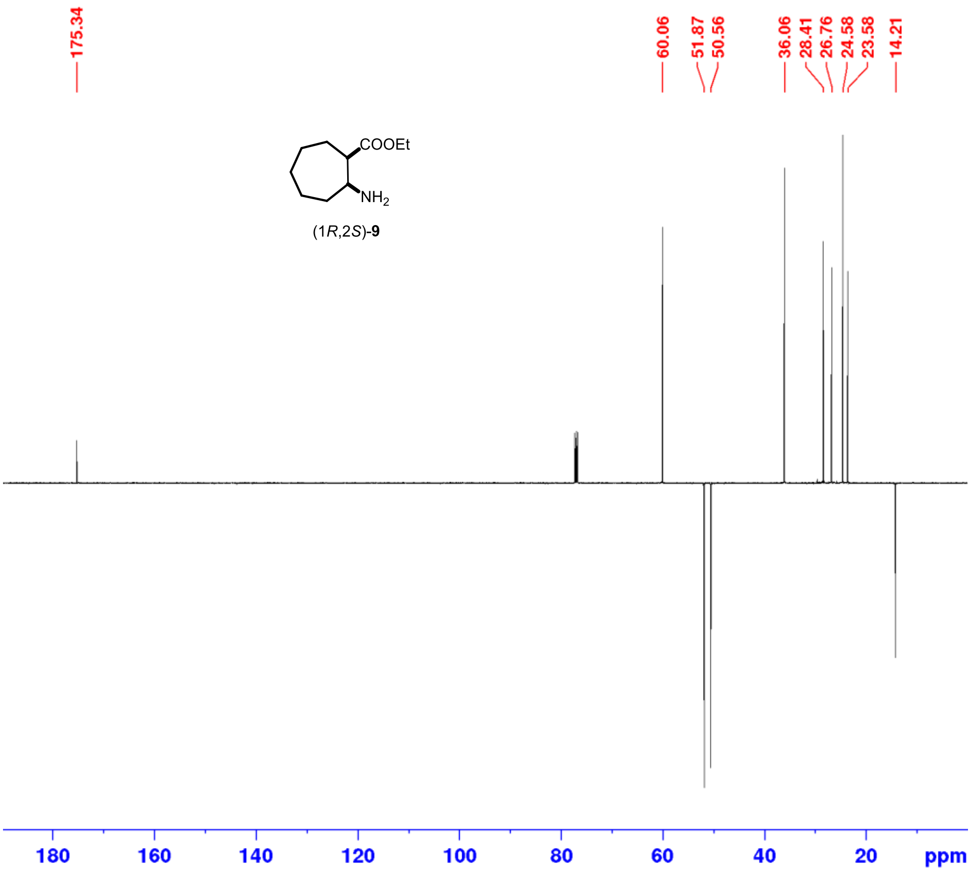

Ethyl (1*R*,2*S*)-2-aminocyclooocanecarboxylate (**13**)

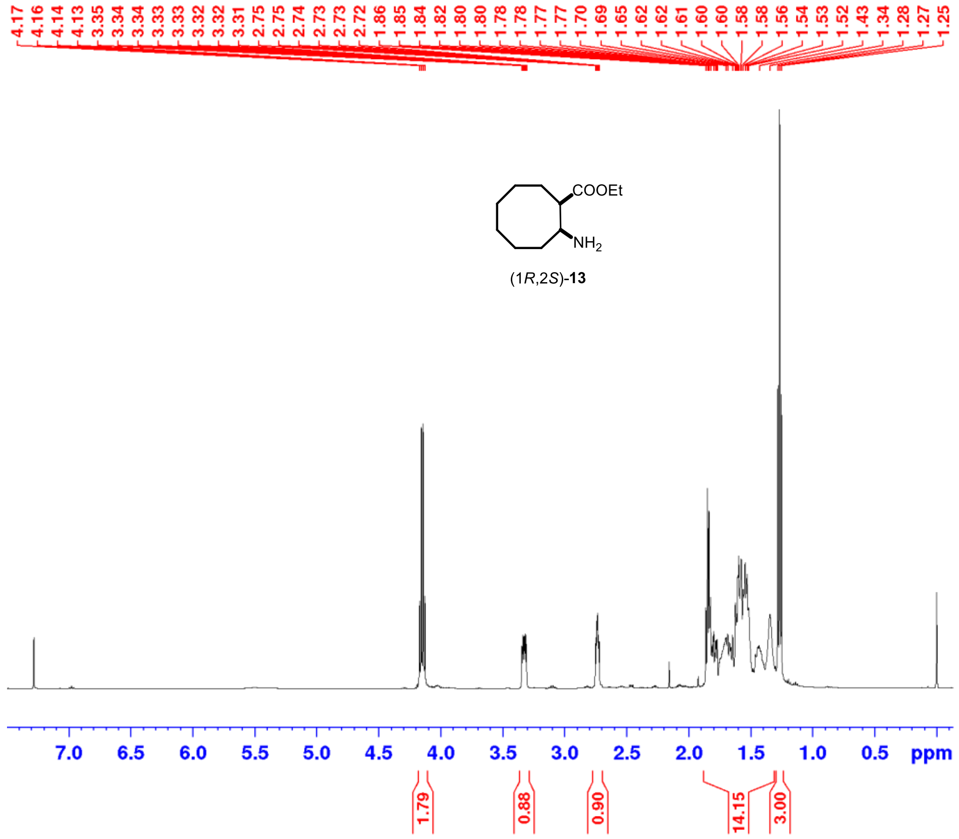

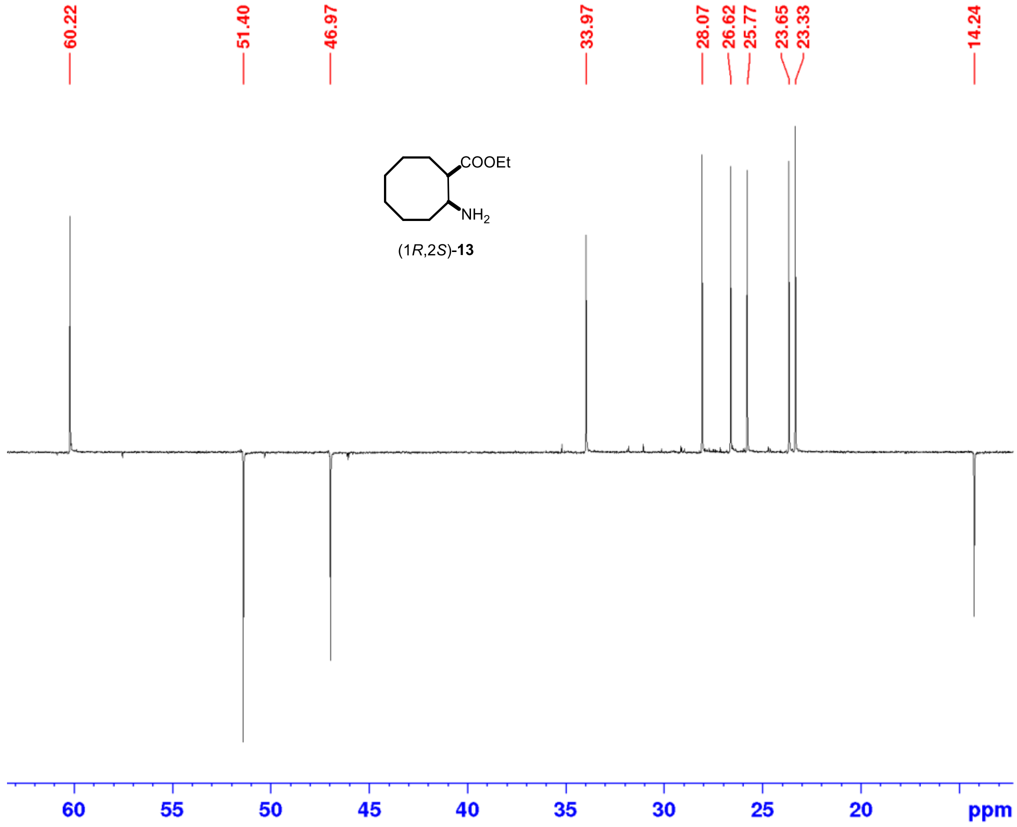

2. ^{1}H- and ^{13}C-NMR spectra of amino acid enantiomers (1*S*,2*R*)-14-17

(1*S*,2*R*)-2-aminocyclopentanecarboxylic acid (**14**)

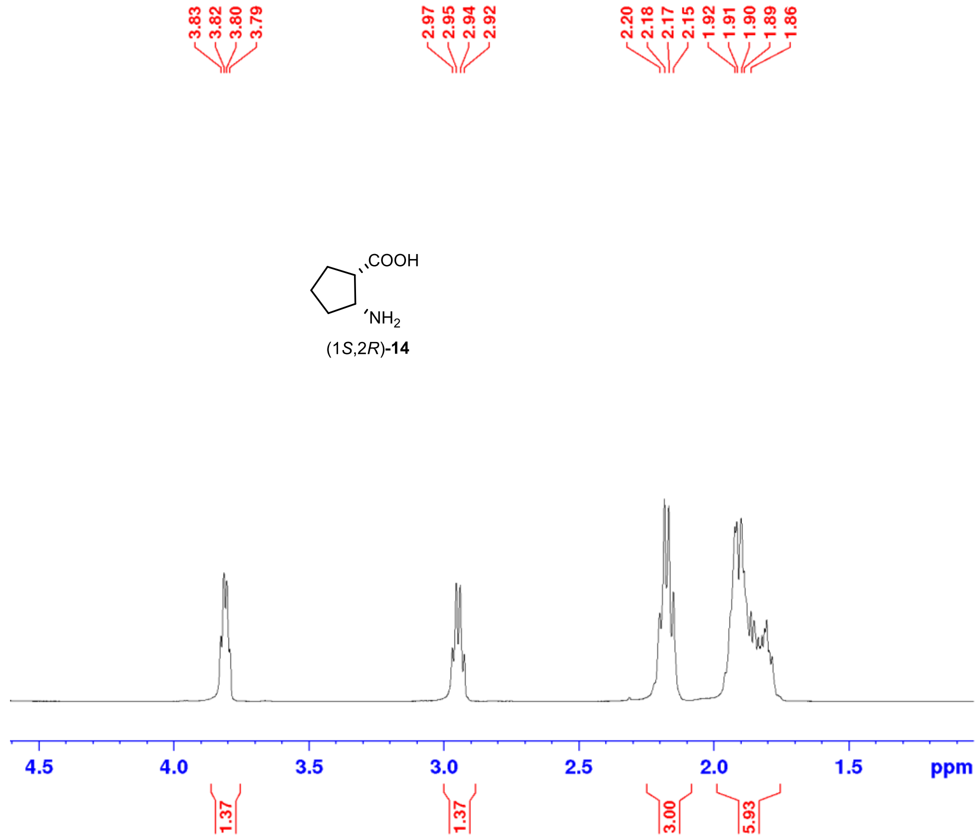

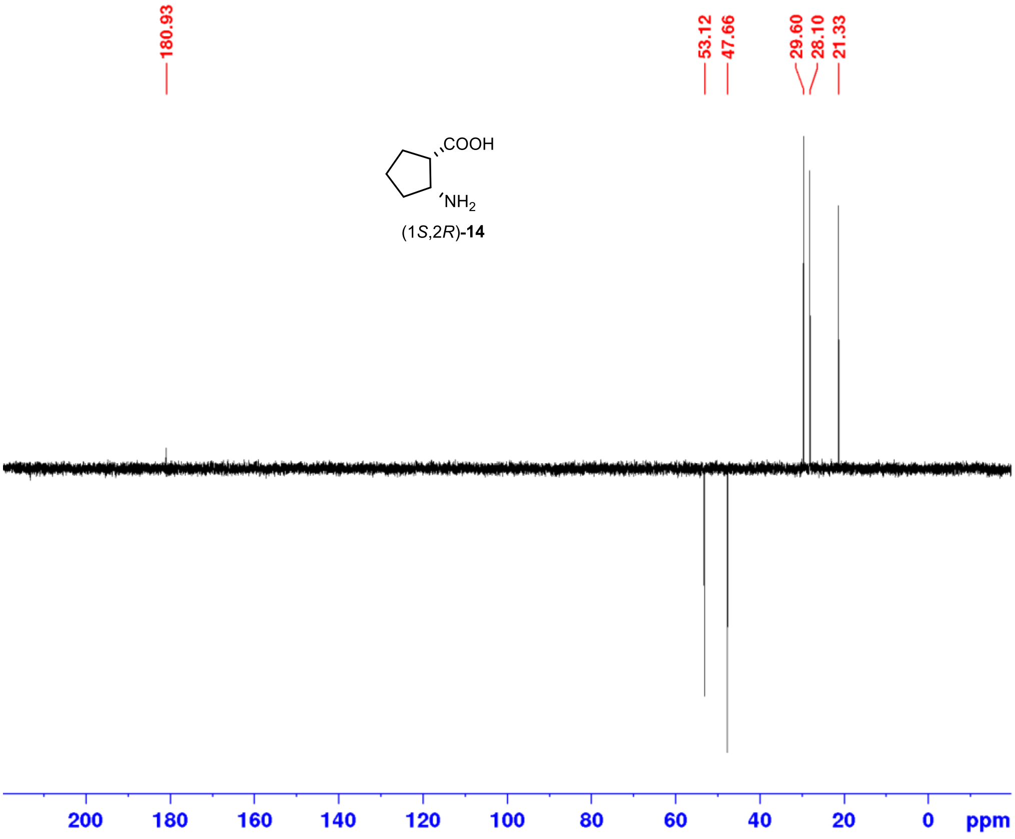

(1*S*,2*R*)-2-aminocyclohexanecarboxylic acid (**15**)

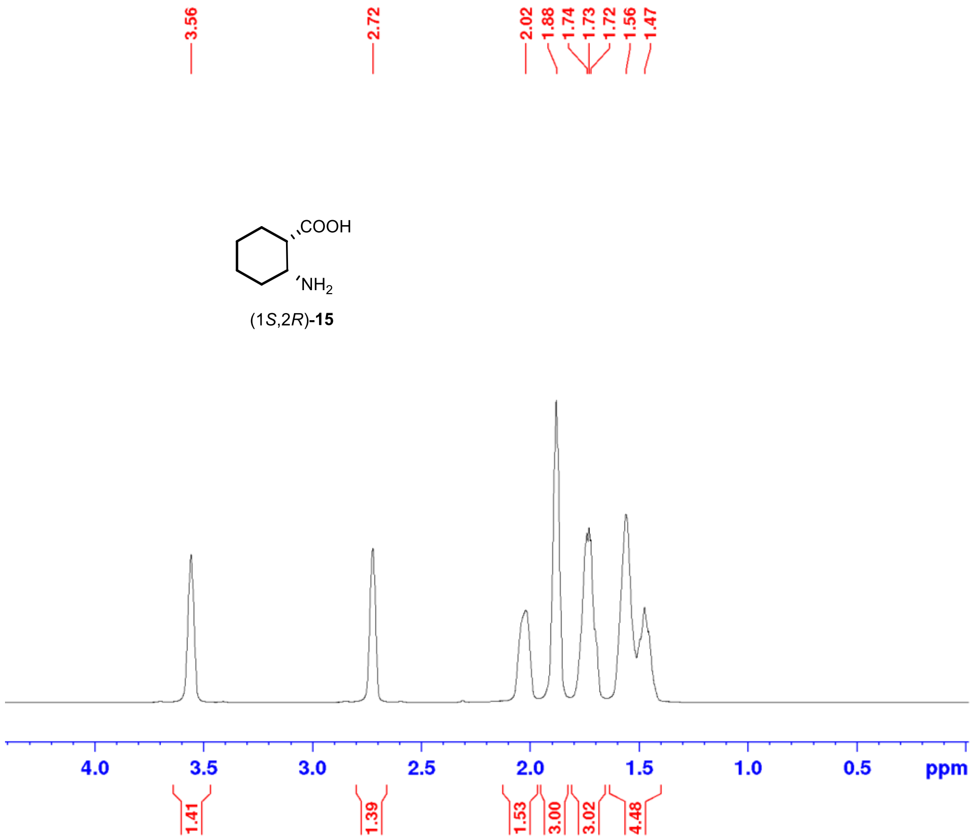

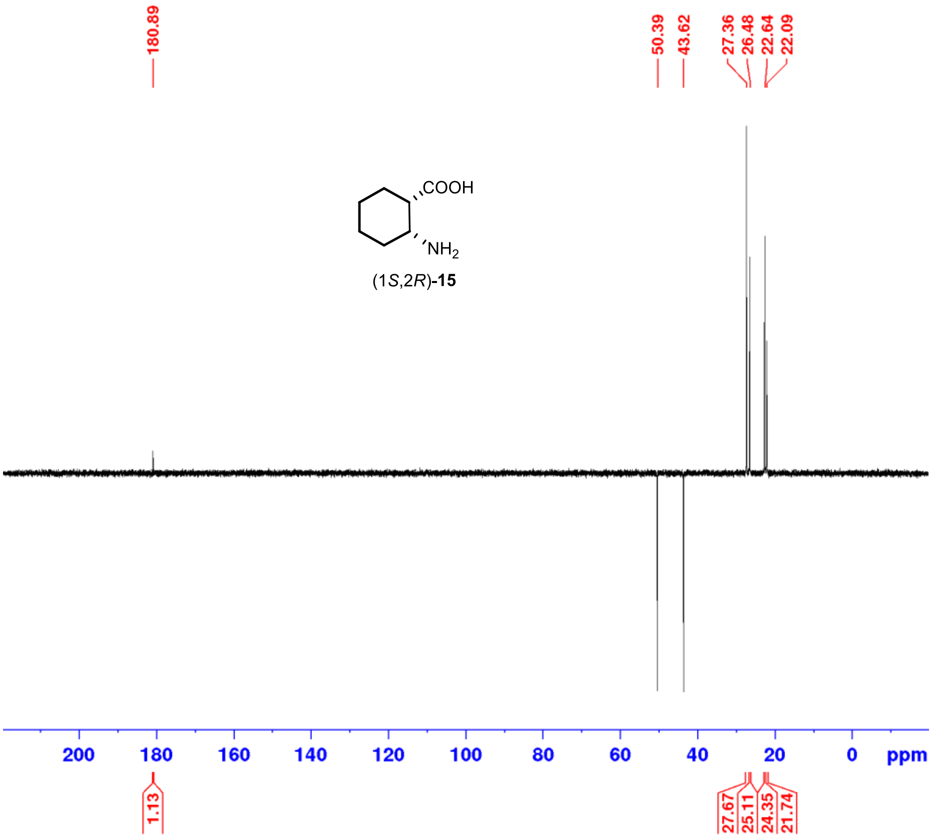

(1*S*,2*R*)-2-aminocycloheptanecarboxylic acid (**16**)

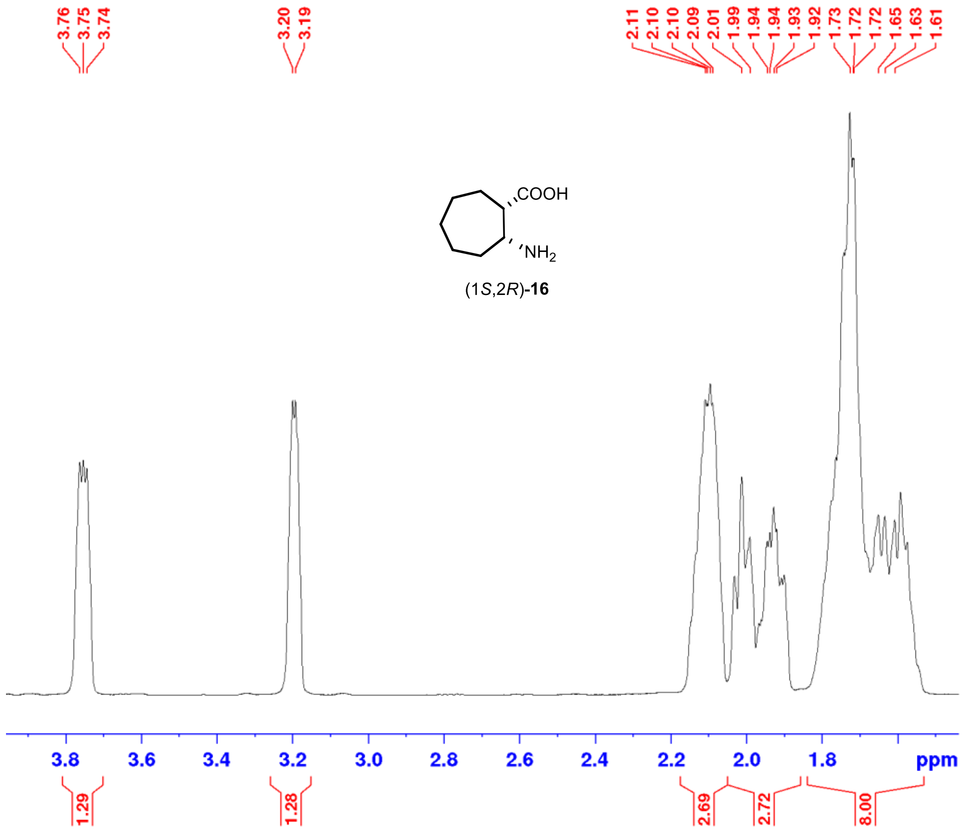

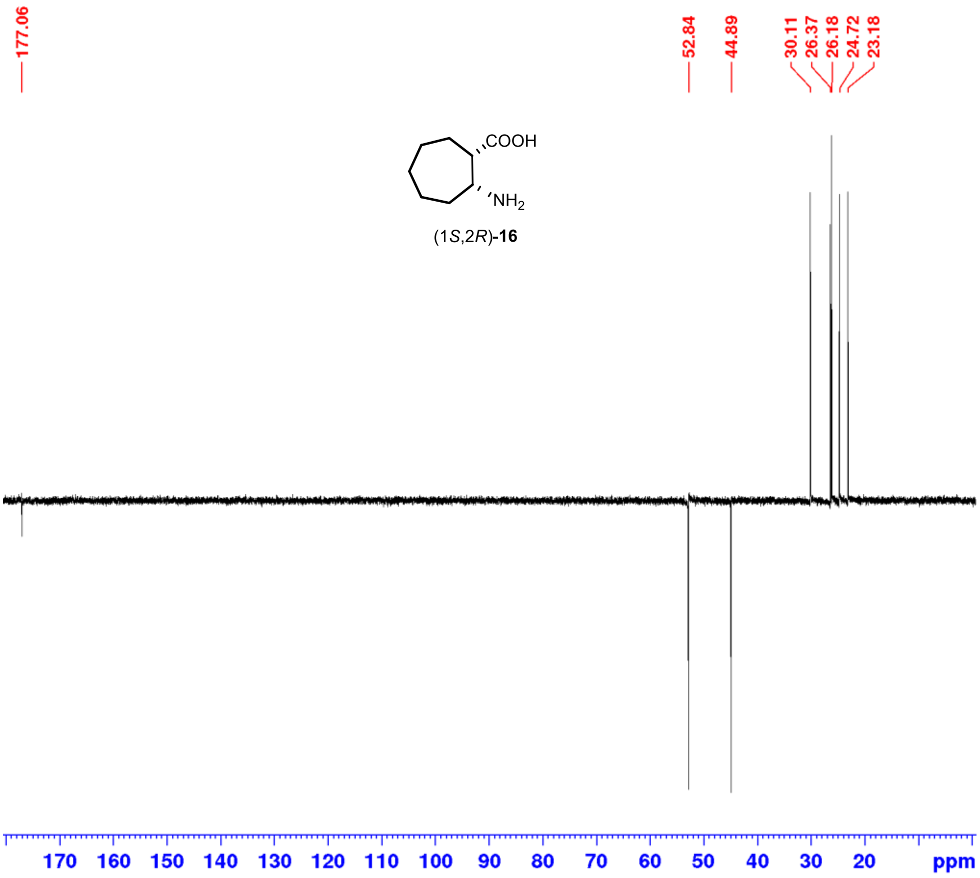

(1*S*,2*R*)-2-aminocyclooctanecarboxylic acid (**17**)

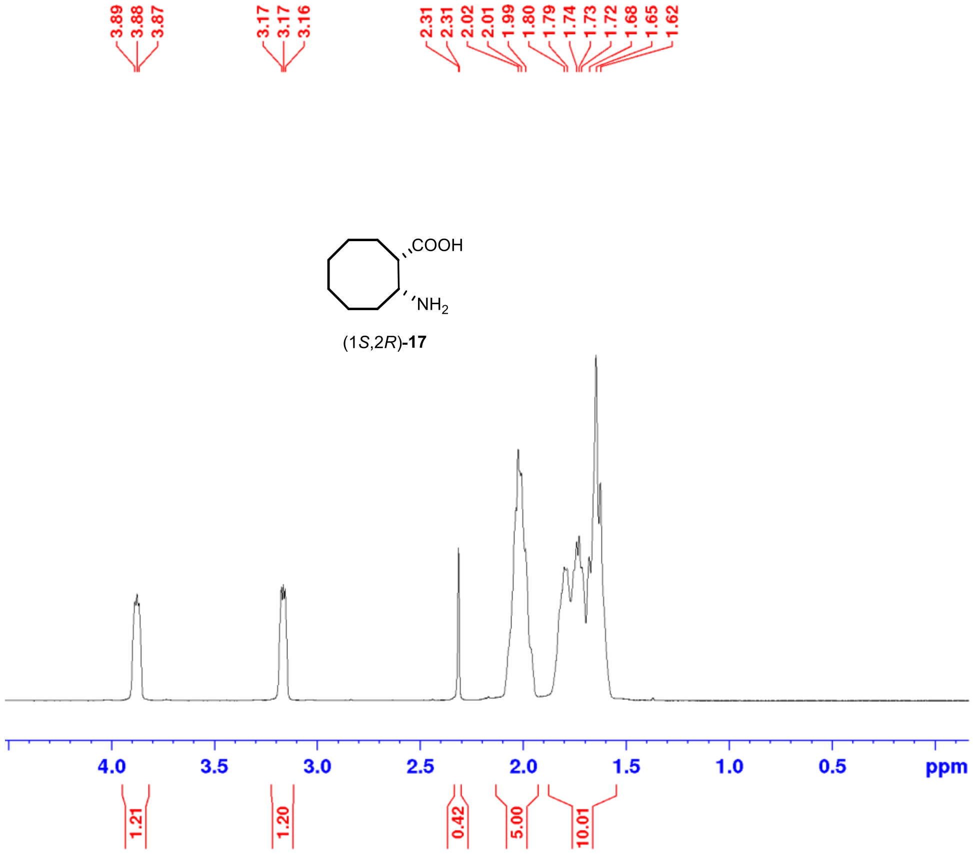

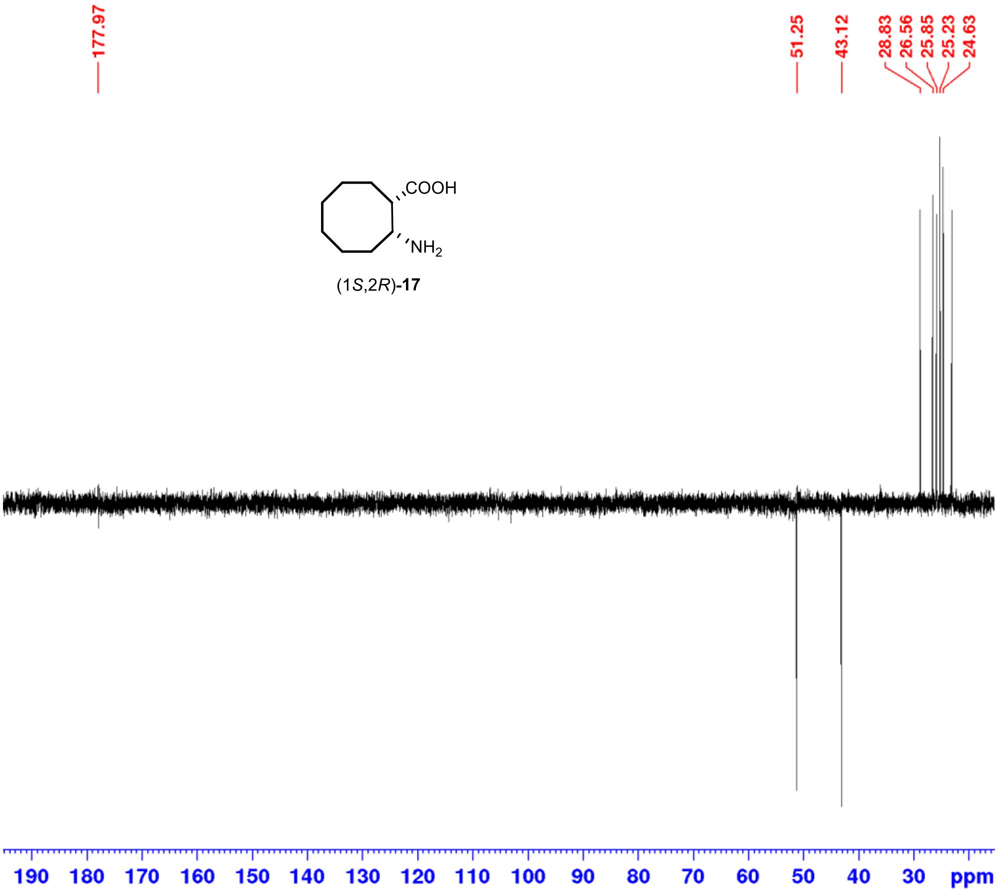

3. GC Chromatograms of ester enantiomers 7-9 and 13:

GC Chromatogram of ethyl-2-aminocyclopentanecarboxylate (**7**): retention times (min) for (1*R*,2*S*)-**7**:13.887 (antipode: 14.331): GC equipped with a Chirasil-L-Val column (25m, 0.25mm, 0.12µm), 80 °C for 5 min → 150 °C (temperature rise 15 °C min^{-1}), 15 psi

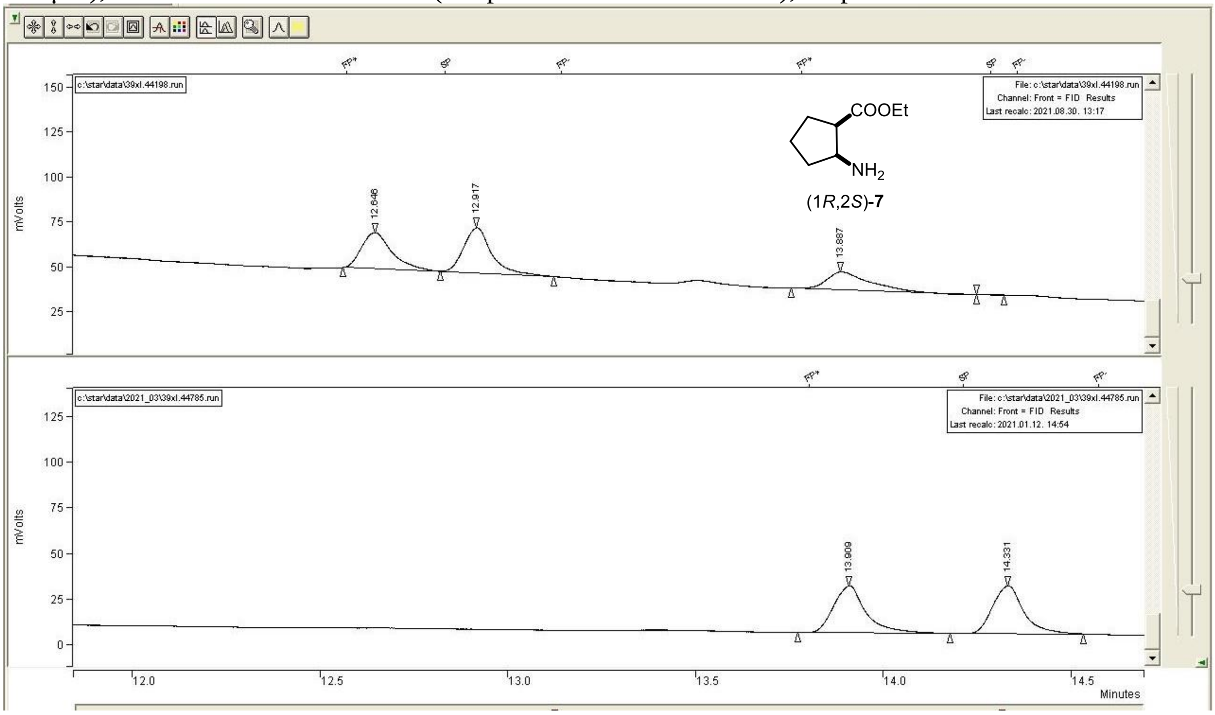

GC Chromatogram of ethyl-2-aminocyclohexanescarboxylate (**8**): retention times (min) for (1*R*,2*S*)-**8**: 16.058 (antipode: 16.316): GC equipped with a Chirasil-L-Val column (25m, 0.25mm, 0.12µm), 80 °C for 5 min → 150 °C (temperature rise 15 °C min^{-1}), 15 psi

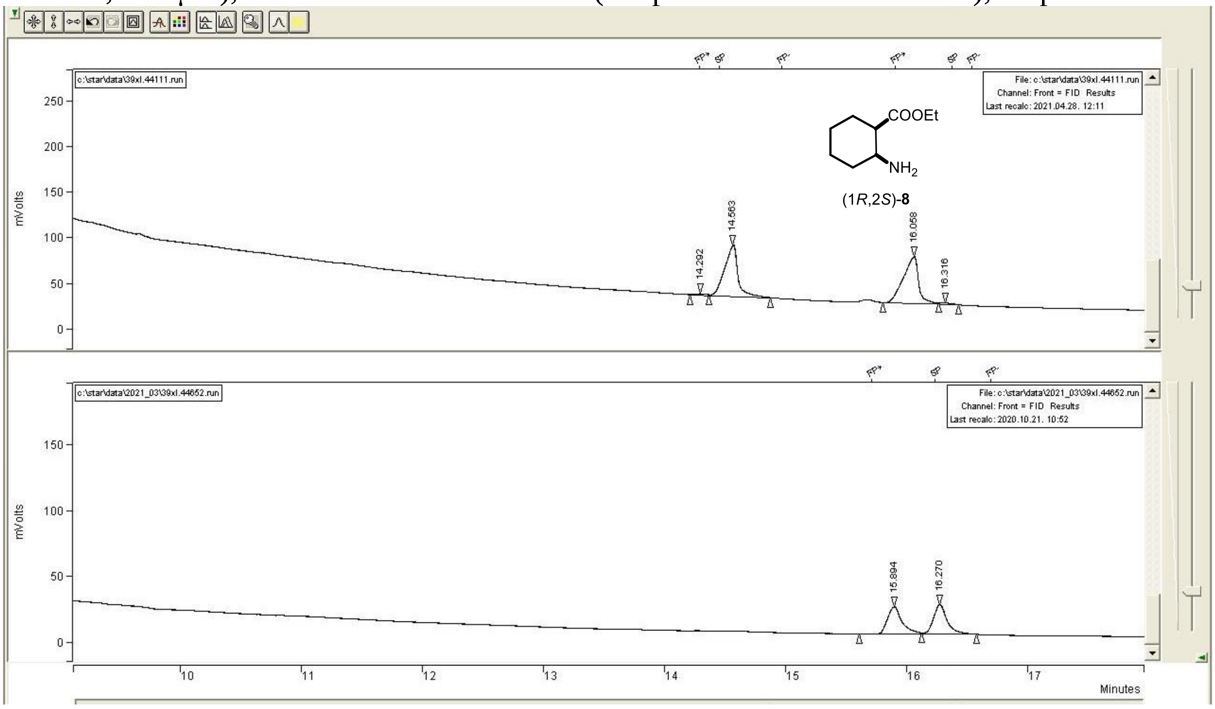

GC Chromatogram of ethyl-2-aminocycloheptanecarboxylate (**9**): retention times (min) for (1*R*,2*S*)-**9**: 40.975 (antipode: 41.865), GC equipped with a Chirasil-L-Val column (25m, 0.25mm, 0.12μm), 50 °C for 5 min → 140 °C (temperature rise 10 °C min^{-1}), 10 psi

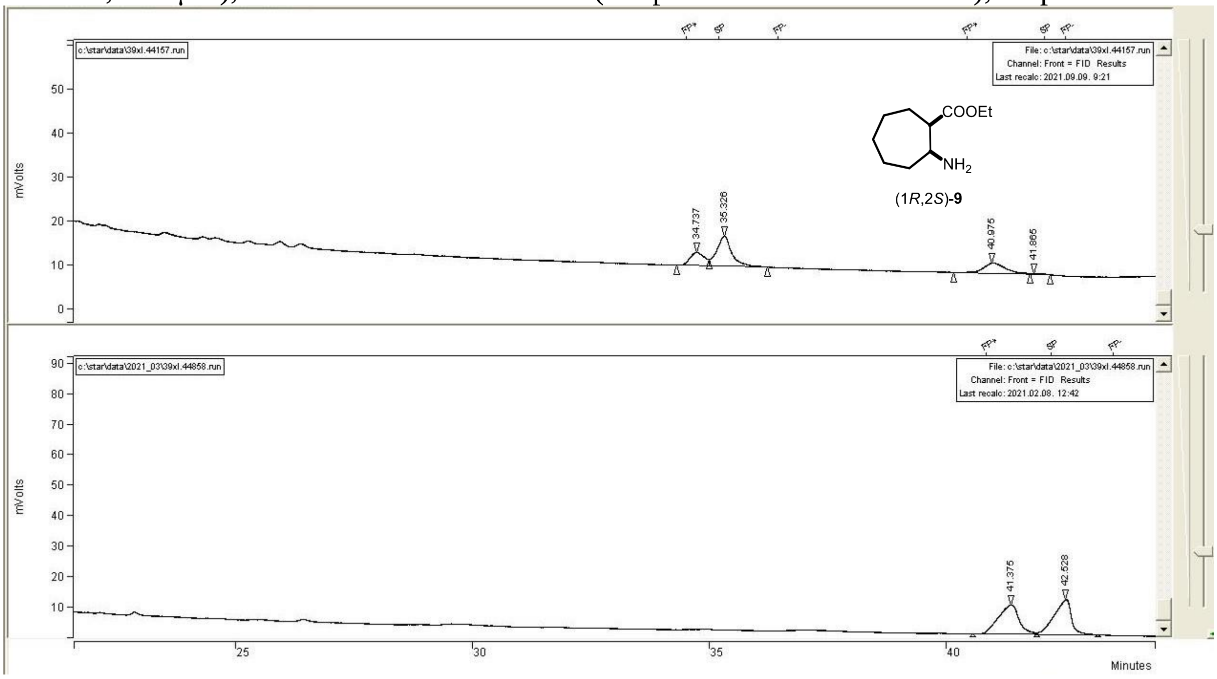

GC Chromatogram of ethyl-2-aminocyclooctanecarboxylate (**13**): retention times (min) for (1*R*,2*S*)-**13**: 57.405 (antipode: 59.240), GC equipped with a Chirasil-L-Val column (25m, 0.25mm, 0.12μm), 50 °C for 5 min → 140 °C (temperature rise 10 °C min^{-1}), 10 psi

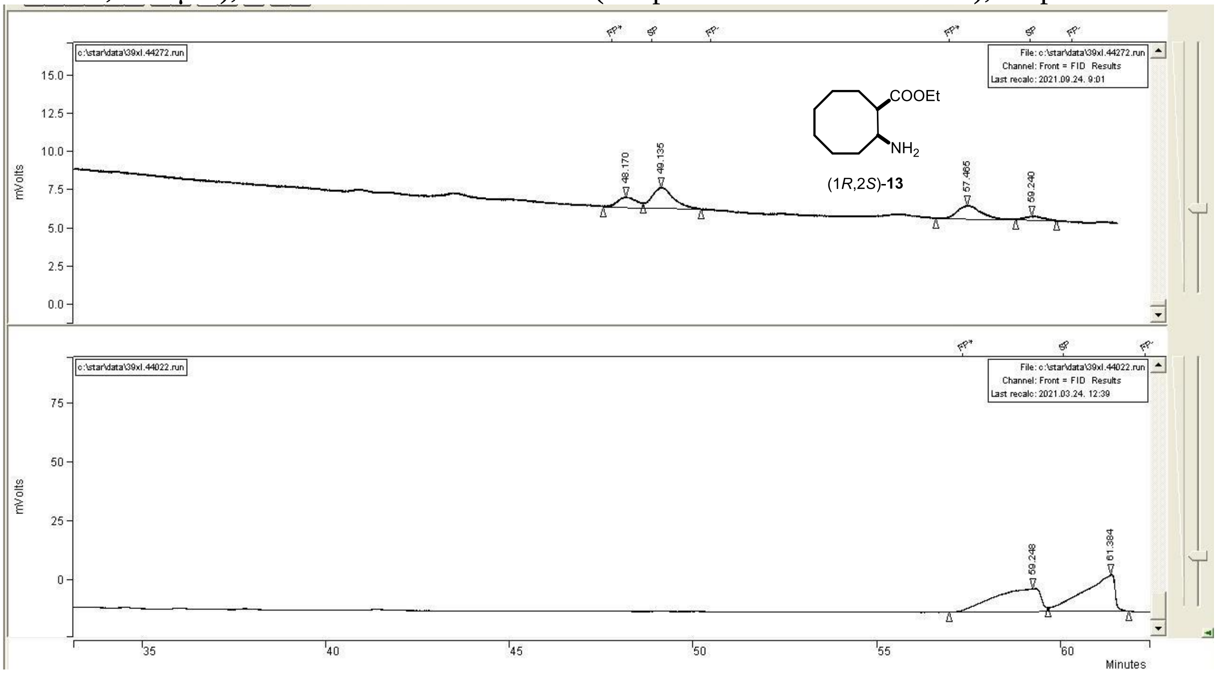

4. GC Chromatograms of amino acid enantiomers 14-17:

GC Chromatograms of 2-aminocyclopentanecarboxylic acid (**14**): retention times (min) for (1*S*,2*R*)-**14**: 12.963 (antipode: 12.674), GC equipped with a Chirasil-L-Val column (25m, 0.25mm, 0.12μm), 80 °C for 5 min → 150 °C (temperature rise 15 °C min^{-1}), 15 psi

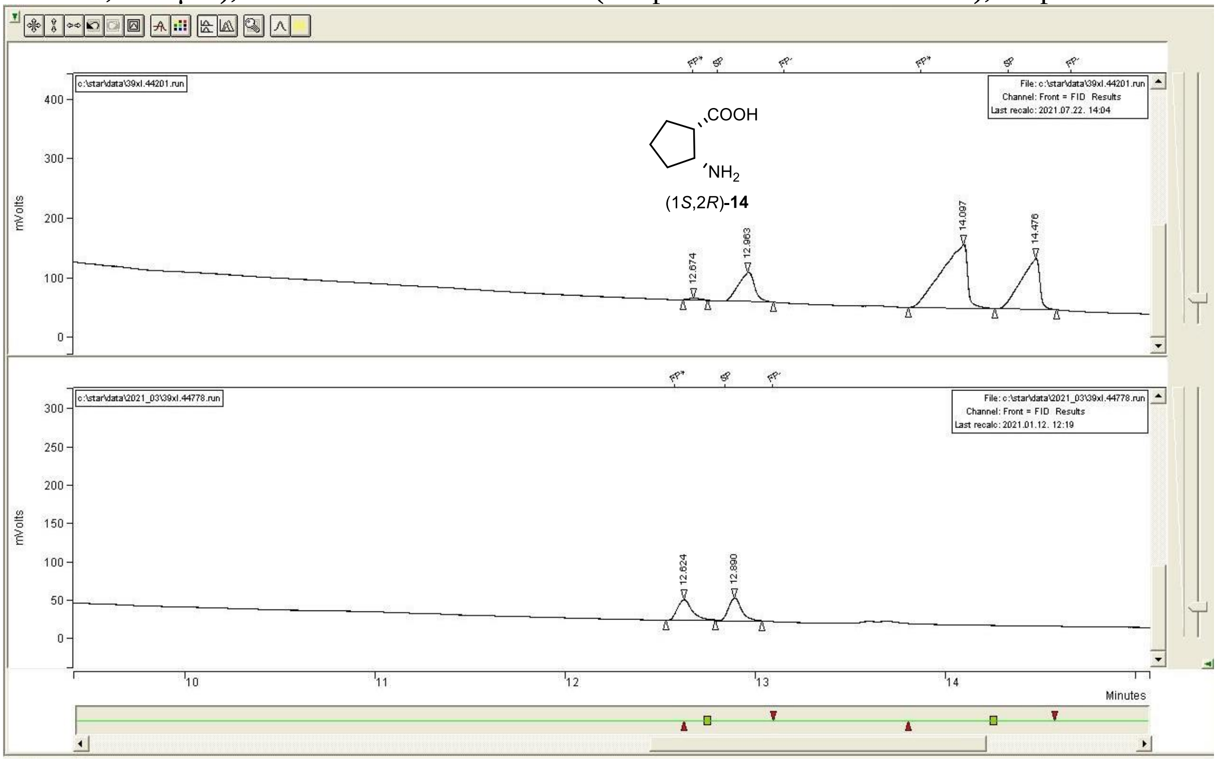

GC Chromatograms of 2-aminocyclohexanecarboxylic acid (**15**): retention times (min) for (1*S*,2*R*)-**15**: 14.563 (antipode: 14.292), GC equipped with a Chirasil-L-Val column (25m, 0.25mm, 0.12μm), 80 °C for 5 min → 150 °C (temperature rise 15 °C min^{-1}), 15 psi

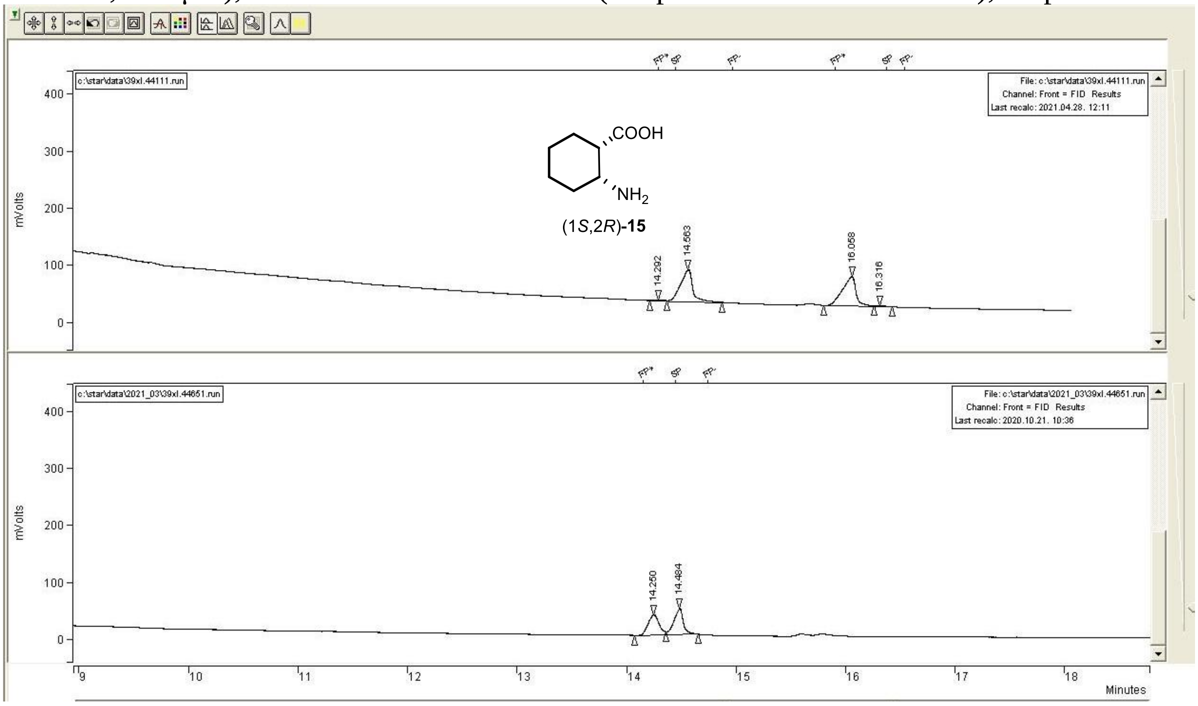

GC Chromatograms of 2-aminocycloheptanecarboxylic acid (**16**): retention times (min) for (1*S*,2*R*)-**16**: 35.641 (antipode: 34.869) GC equipped with a Chirasil-L-Val column (25m, 0.25mm, 0.12µm), 50 °C for 5 min → 140 °C (temperature rise 10 °C min^{-1}), 10 psi

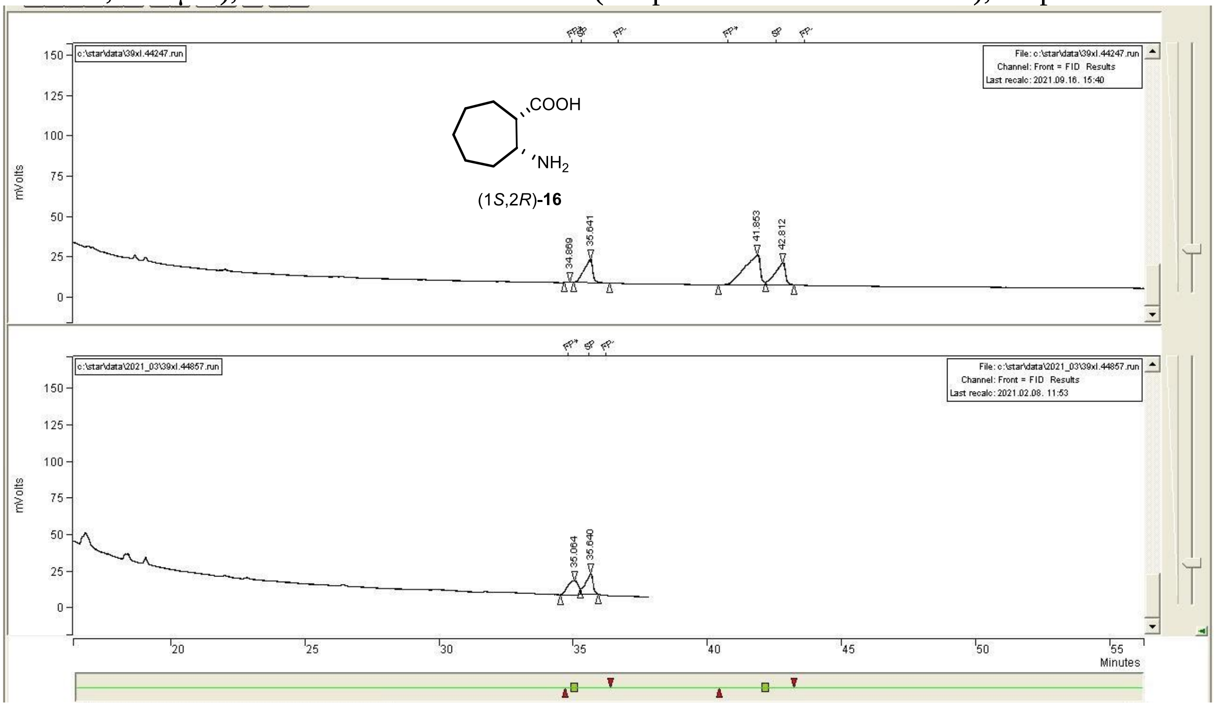

GC Chromatograms of 2-aminocyclooctanecarboxylic acid (**17**): retention times (min) for (1*S*,2*R*)-**17**: 49.309 (antipode: 48.819), GC equipped with a Chirasil-L-Val column (25m, 0.25mm, 0.12µm), 50 °C for 5 min → 140 °C (temperature rise 10 °C min^{-1}), 10 psi

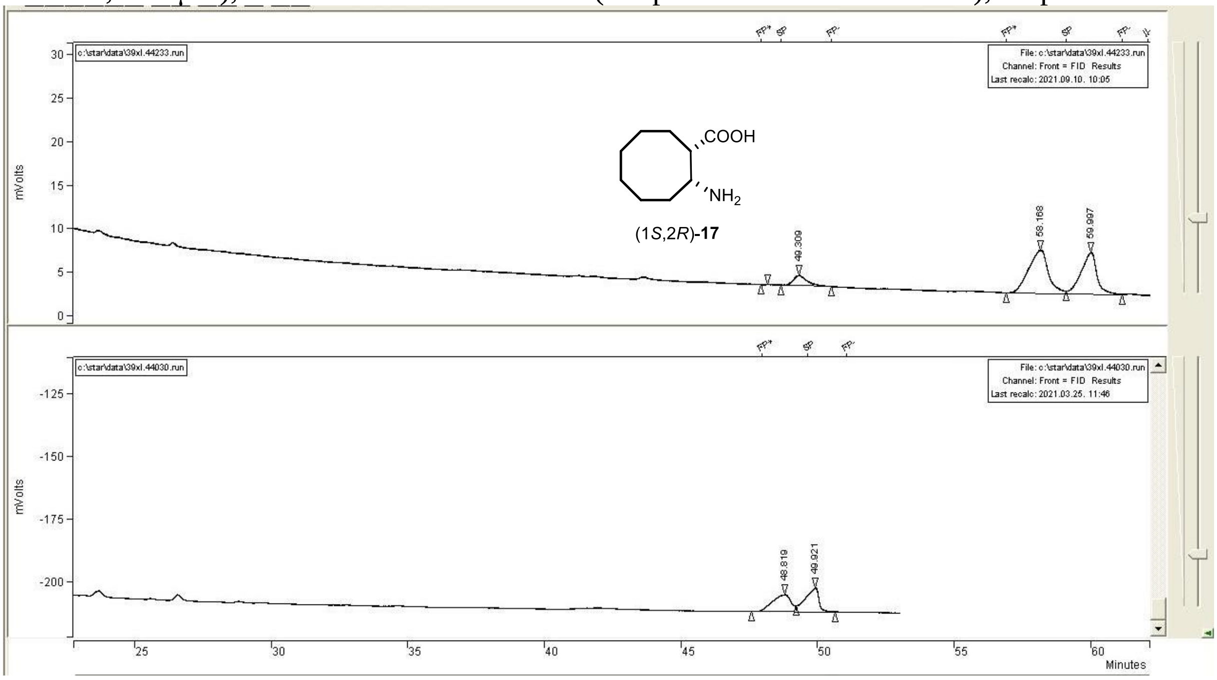

5. HRMS (ESI) spectra of ester enantiomers (1*R*,2*S*)-7-9 and (1*R*,2*S*)-13

HRMS spectrum of ethyl (1*R*,2*S*)-2-aminocyclopentanecarboxylate (**7**)

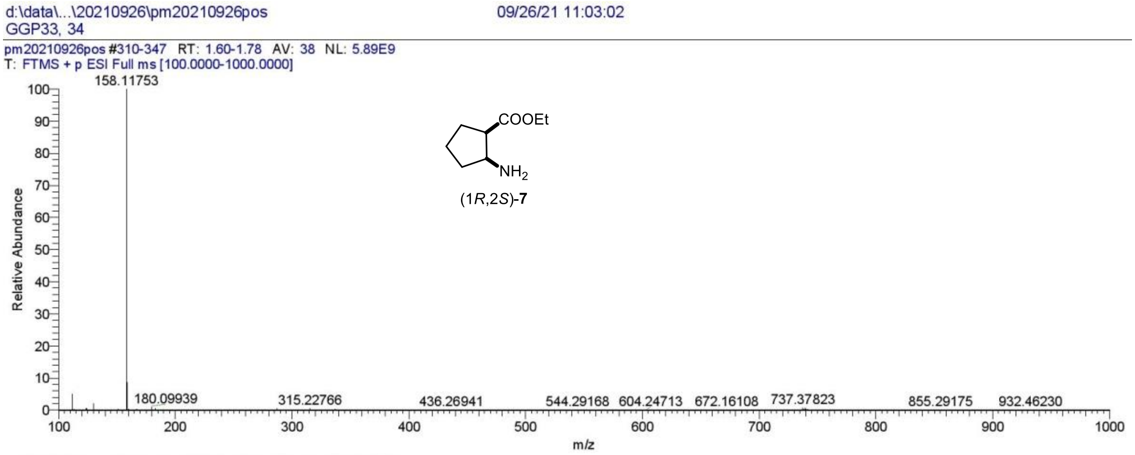

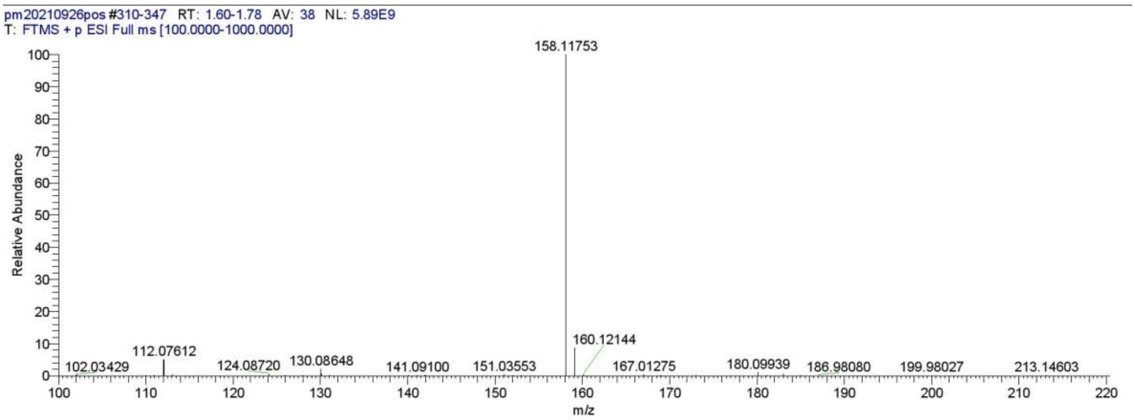

HRMS spectrum of ethyl (1*R*,2*S*)-2-aminocyclohexanecarboxylate (**8**)

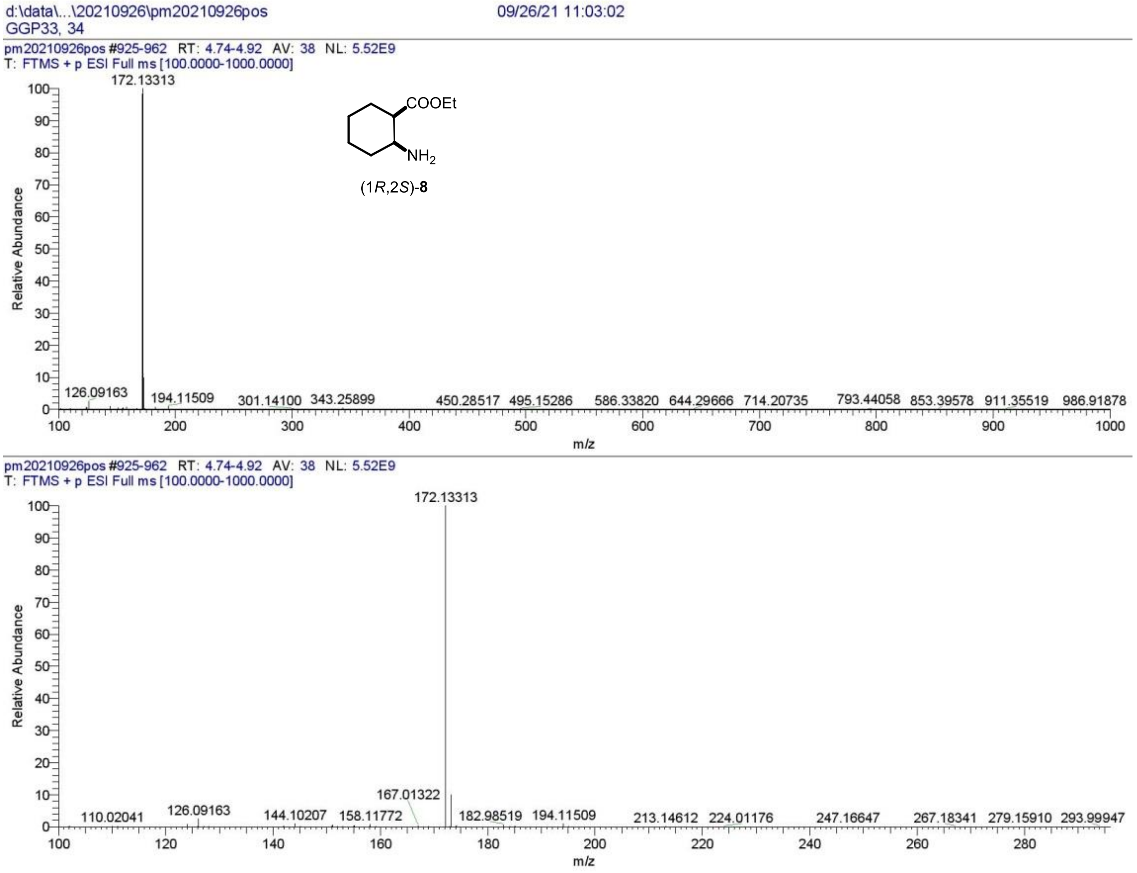

HRMS spectrum of ethyl (1*R*,2*S*)-2-aminocycloheptanecarboxylate (**9**)

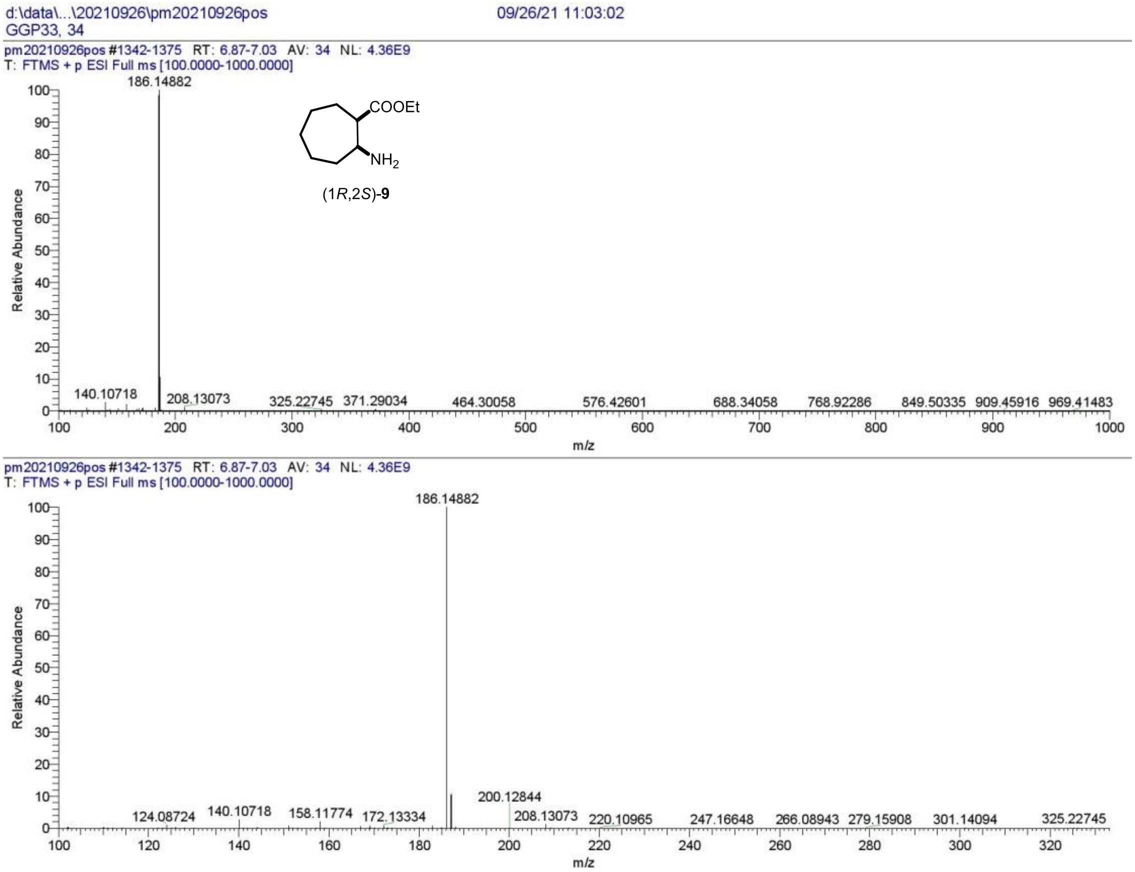

HRMS spectrum of ethyl (1*R*,2*S*)-2-aminocyclooctanecarboxylate (**13**)

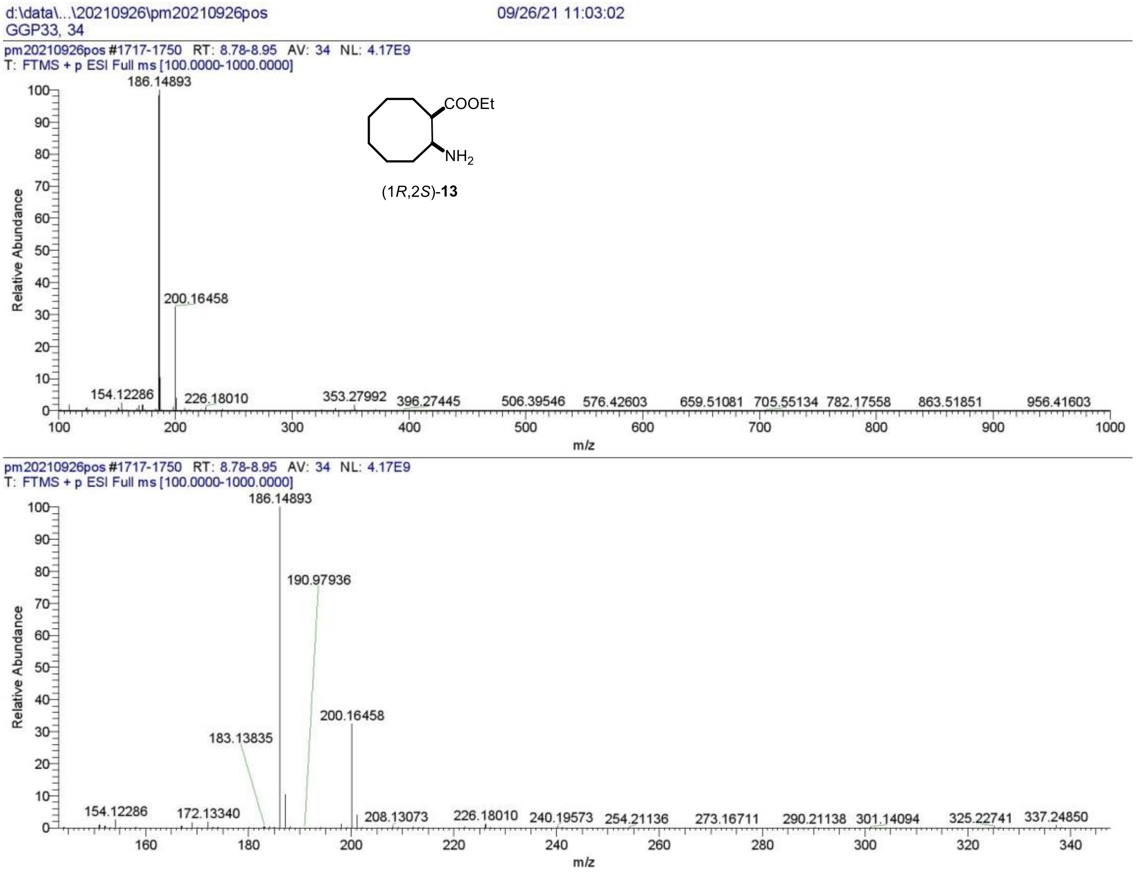

6. HRMS (ESI) spectra of amino acid enantiomers (1*S*,2*R*)-14-17

HRMS spectrum of (1*S*,2*R*)-2-aminocyclopentanecarboxylic acid (**14**)

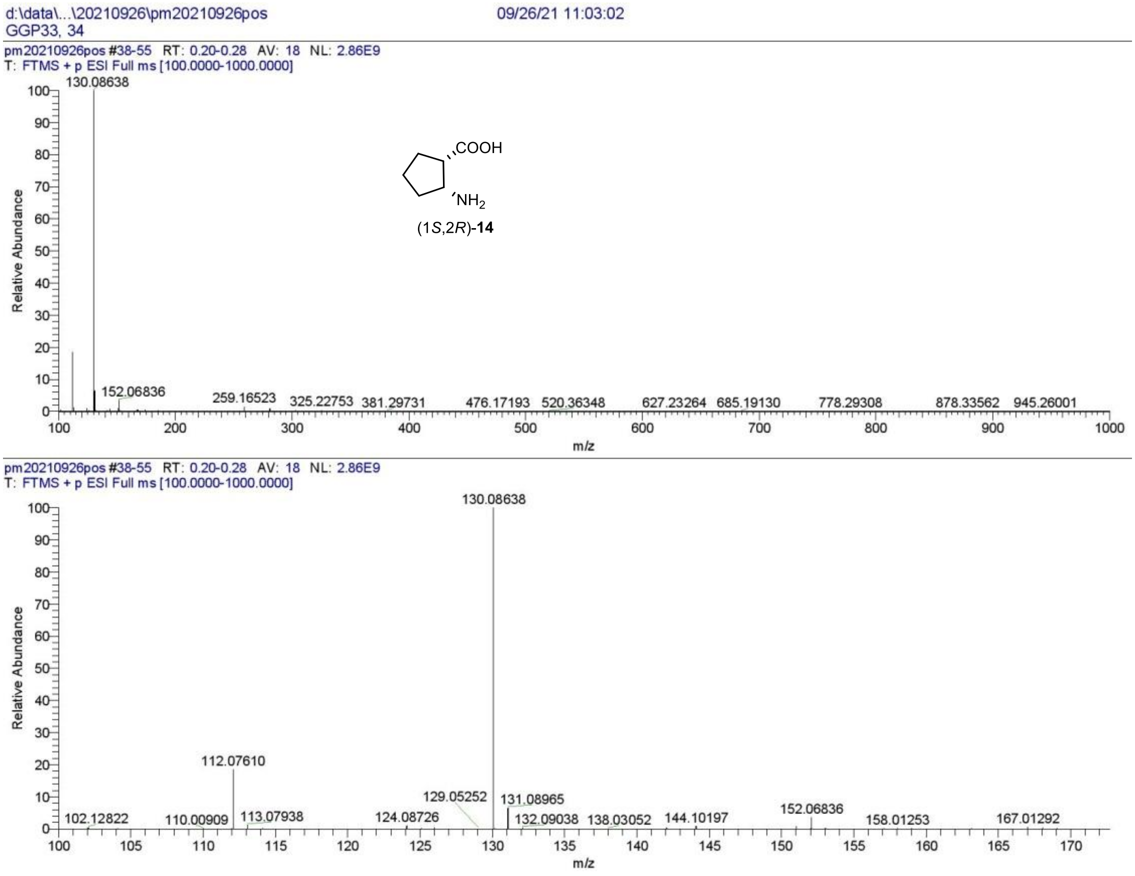

HRMS spectrum of (1*S*,2*R*)-2-aminocyclohexanecarboxylic acid (**15**)

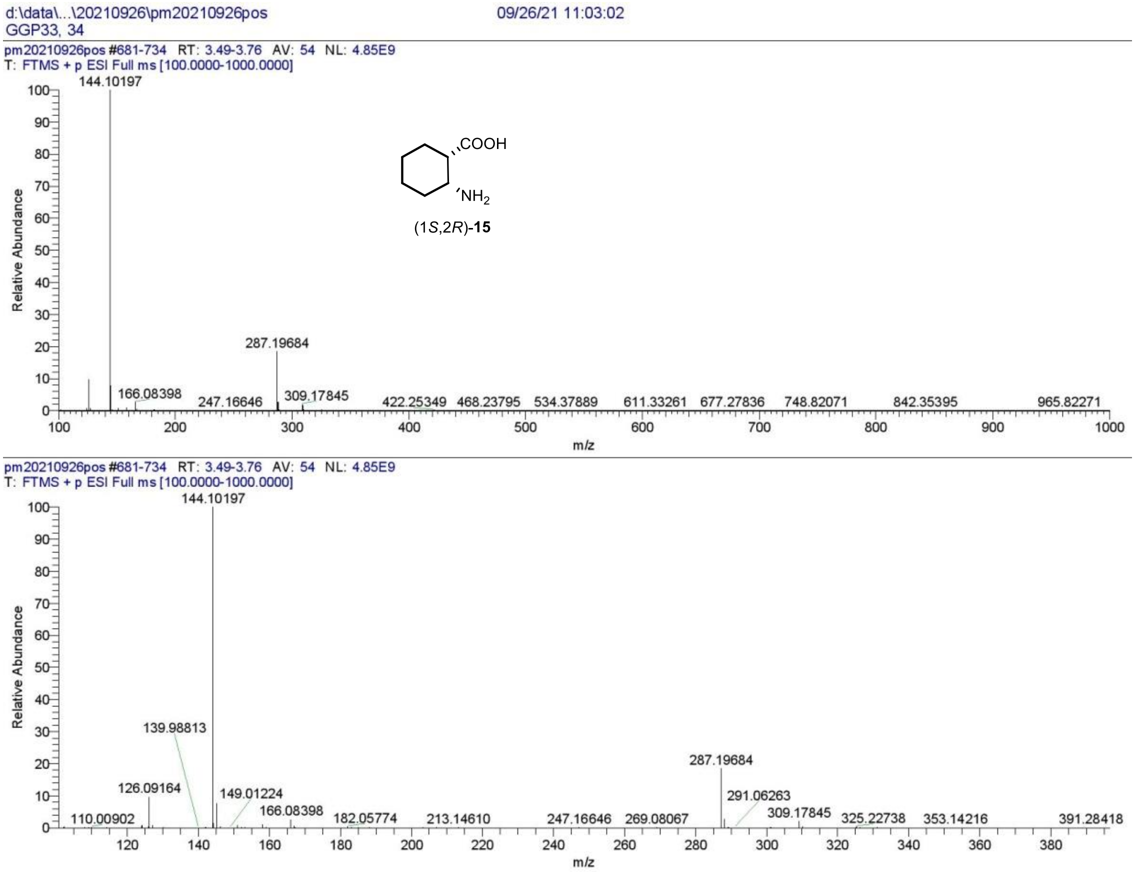

HRMS spectrum of (1*S*,2*R*)-2-aminocycloheptanecarboxylic acid (**16**)

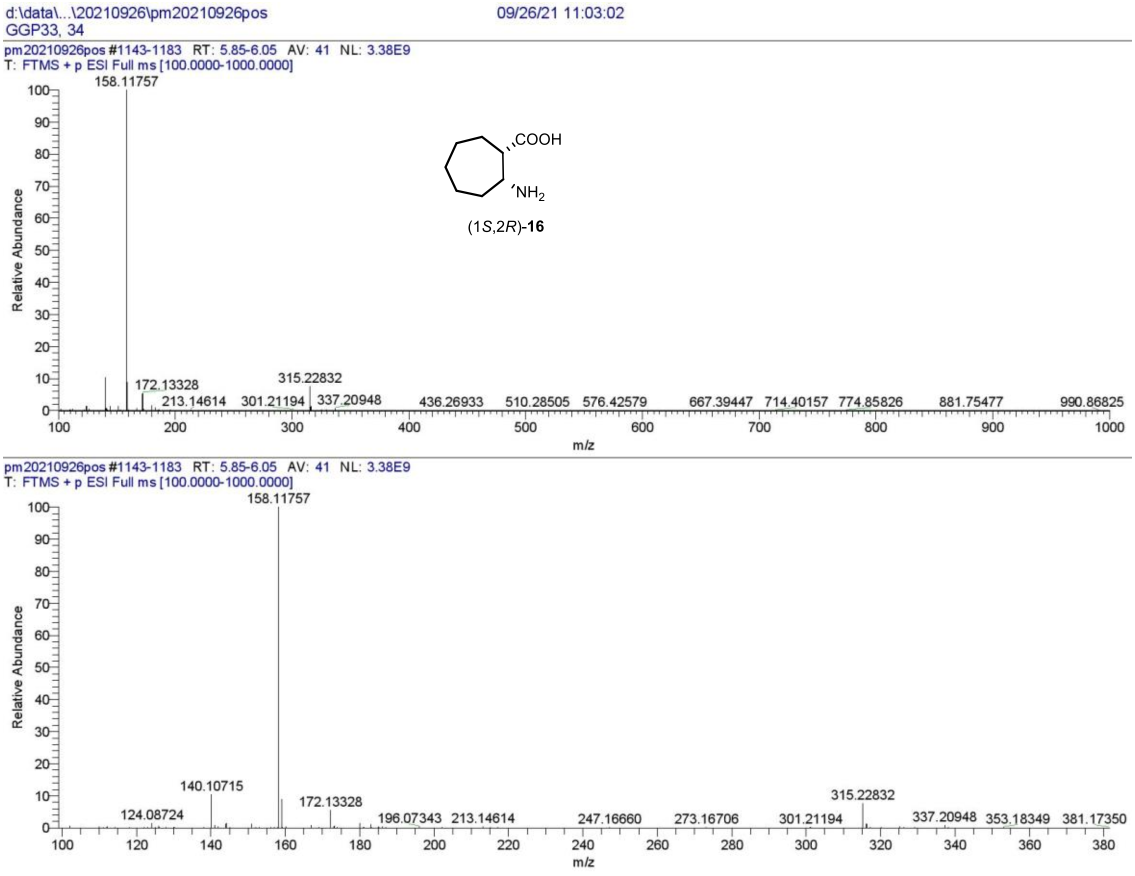

HRMS spectrum of (1*S*,2*R*)-2-aminocyclooctanecarboxylic acid (**17)**

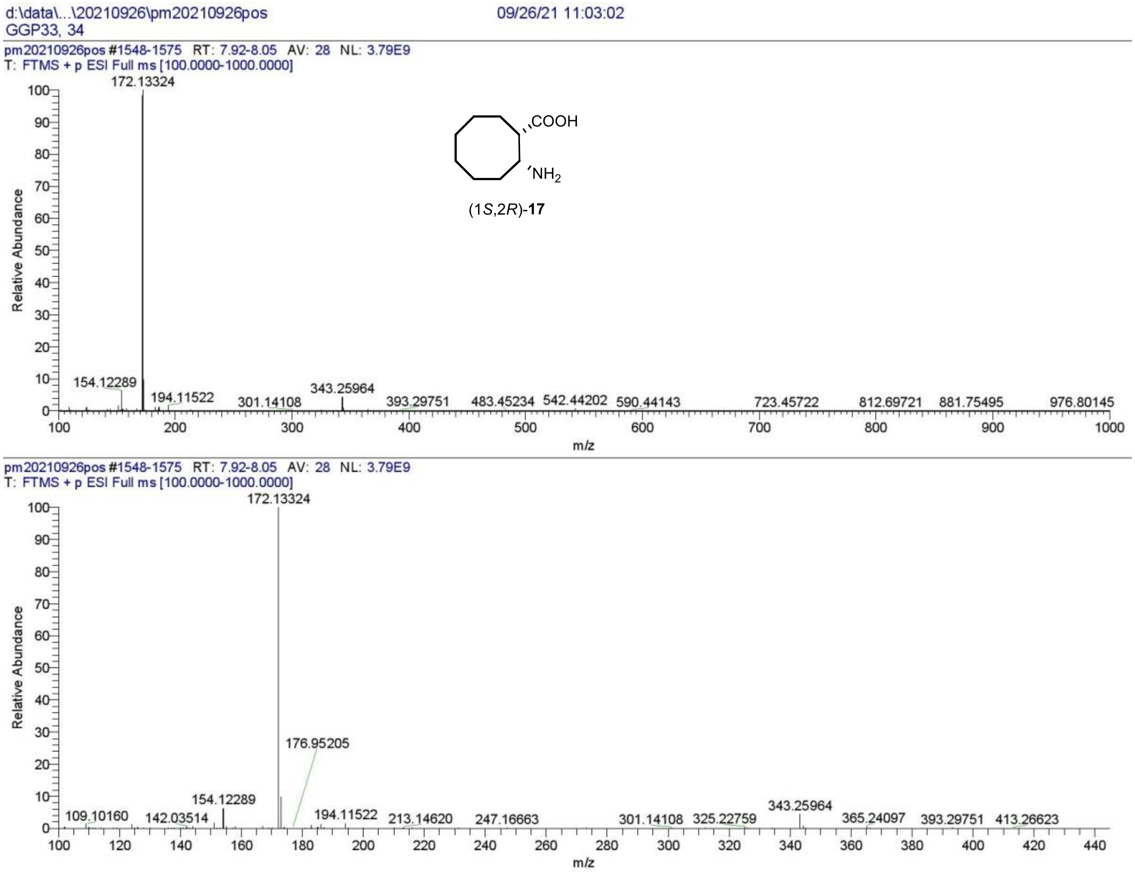

Supplement: Supplementary file 1 [file molecules-27-02600-s001.zip › molecules-1660343-supplementary.pdf]
